# Reactive halogens increase the global methane lifetime and radiative forcing in the 21st century

**DOI:** 10.1038/s41467-022-30456-8

**Published:** 2022-05-19

**Authors:** Qinyi Li, Rafael P. Fernandez, Ryan Hossaini, Fernando Iglesias-Suarez, Carlos A. Cuevas, Eric C. Apel, Douglas E. Kinnison, Jean-François Lamarque, Alfonso Saiz-Lopez

**Affiliations:** 1 Department of Atmospheric Chemistry and Climate, Institute of Physical Chemistry Rocasolano, CSIC, Madrid, 28006 Spain; 2 Institute for Interdisciplinary Science (ICB), National Research Council (CONICET), FCEN-UNCuyo, Mendoza, Argentina; 3 Lancaster Environment Centre, Lancaster University, Lancaster, UK; 4 Deutsches Zentrum für Luft- und Raumfahrt (DLR), Institut für Physik der Atmosphäre, Oberpfaffenhofen, Germany; 5 Atmospheric Chemistry Observations & Modeling Laboratory, National Center for Atmospheric Research, Boulder, CO USA; 6 Climate and Global Dynamics Laboratory, National Center for Atmospheric Research, Boulder, CO USA

**Keywords:** Atmospheric science, Climate change

## Abstract

CH_4_ is the most abundant reactive greenhouse gas and a complete understanding of its atmospheric fate is needed to formulate mitigation policies. Current chemistry-climate models tend to underestimate the lifetime of CH_4_, suggesting uncertainties in its sources and sinks. Reactive halogens substantially perturb the budget of tropospheric OH, the main CH_4_ loss. However, such an effect of atmospheric halogens is not considered in existing climate projections of CH_4_ burden and radiative forcing. Here, we demonstrate that reactive halogen chemistry increases the global CH_4_ lifetime by 6–9% during the 21st century. This effect arises from significant halogen-mediated decrease, mainly by iodine and bromine, in OH-driven CH_4_ loss that surpasses the direct Cl-induced CH_4_ sink. This increase in CH_4_ lifetime helps to reduce the gap between models and observations and results in a greater burden and radiative forcing during this century. The increase in CH_4_ burden due to halogens (up to 700 Tg or 8% by 2100) is equivalent to the observed atmospheric CH_4_ growth during the last three to four decades. Notably, the halogen-driven enhancement in CH_4_ radiative forcing is 0.05 W/m^2^ at present and is projected to increase in the future (0.06 W/m^2^ by 2100); such enhancement equals ~10% of present-day CH_4_ radiative forcing and one-third of N_2_O radiative forcing, the third-largest well-mixed greenhouse gas. Both direct (Cl-driven) and indirect (via OH) impacts of halogens should be included in future CH_4_ projections.

## Abstract (web summary)

Methane is a powerful greenhouse gas and previous studies focus on its sources with less attention on the loss. Here the authors show that reactive halogen species, not considered in climate projections, significantly reduces the methane loss, increasing its lifetime, burden, and radiative forcing.

## Introduction

CH_4_ is a greenhouse gas (GHG) with the second largest contribution to climate radiative forcing (RF) after CO_2_ (ref. ^1^). Unlike the inert gas CO_2_, CH_4_ actively participates in atmospheric chemistry. A better understanding of the global CH_4_ budget (i.e., sources and sinks) is vital to constrain its atmospheric levels and RF in the 21st century, as well as to guide climate mitigation efforts, e.g., policies to fulfill the Paris agreement^1,2^. Previous research on CH_4_ has reported various types and strengths of natural and anthropogenic emission sources and trends^3–6^. It is also well established that the direct reaction with the OH radical (Eq. 1), the principal atmospheric oxidant, is the dominant chemical sink of CH_4_ in the atmosphere. Chlorine atoms (Cl) provide an additional sink (Eq. 2)^7–9^. Due to the difficulty in directly observing OH and Cl, the quantification of the global abundance of these radicals, as well as their consequent chemical impacts, depends heavily on model simulations^4^. However, chemistry-climate models vary strongly in their simulated atmospheric CH_4_ lifetime and, on average, tend to underestimate the CH_4_ lifetime^10–13^, pointing to potential uncertainties in CH_4_ sources or sinks.

1 $${{{{{\rm{OH}}}}}}+{{{{{{\rm{CH}}}}}}}_{4}\to {{{{{{\rm{CH}}}}}}}_{3}{{{{{{\rm{O}}}}}}}_{2}+{{{{{{\rm{H}}}}}}}_{2}{{{{{\rm{O}}}}}}$$

2 $${{{{{\rm{Cl}}}}}}+{{{{{{\rm{CH}}}}}}}_{4}\to {{{{{{\rm{CH}}}}}}}_{3}{{{{{{\rm{O}}}}}}}_{2}+{{{{{\rm{HCl}}}}}}$$

Over the past decades, a large and growing body of observational evidence has demonstrated that the emission and ubiquitous existence of reactive halogen species (RHS; Cl, Br, and I containing species with a lifetime <180 days) in the troposphere, including natural and anthropogenic very short-lived halocarbons, exert a powerful influence on the tropospheric chemical composition^14–22^ (Methods). The photochemical breakdown of chlorine-containing RHS results in the release of chlorine atoms (Eqs. 3–5) which provide a direct chemical sink for CH_4_ in the atmosphere (Eq. 2). However, RHS chemistry in the troposphere also affects the budget of OH: in clean environments (e.g., open ocean, polar regions, free troposphere, etc.), halogen atoms (particularly I and Br) destroy O_3_ (ref. ^17^), the main source of the OH radical^23^, therefore indirectly decreasing the tropospheric OH abundance (Eqs. 6–8); in the polluted regions with high emissions of nitrogen oxides (NO_x_) and volatile organic compounds (VOCs), halogens tend to increase the production of OH (Eqs. 9–12)^24,25^; and halogens also alter the partitioning of HO_x_ (OH and HO_2_) by transforming HO_2_ into OH in all regions (Eqs. 13–14)^20^. While the direct effect of chlorine on CH_4_ loss rate and lifetime (atmospheric chemistry terms) has been quantified by a few studies^7–9,12^, there is only one study that estimated the indirect effect of RHS (via OH) on CH_4_ lifetime for present-day conditions^21^. Therefore, the combined (i.e., direct + indirect) effects of halogens on the CH_4_ RF and atmospheric burden (critical parameters for the climate system) and more importantly their variations under a changing climate during the 21st century have until now remained unknown due to the omission of RHS in the chemistry-climate model projections.

3 $${{{{{\rm{ClX}}}}}}+{hv}\to {{{{{\rm{Cl}}}}}}+{{{{{\rm{X}}}}}}({{{{{\rm{X}}}}}}={{{{{\rm{Cl}}}}}};{{{{{\rm{Br}}}}}};{{{{{\rm{and}}}}}}\,{{{{{\rm{I}}}}}})$$

4 $${{{{{{\rm{ClNO}}}}}}}_{2}+{hv}\to {{{{{\rm{Cl}}}}}}+{{{{{{\rm{NO}}}}}}}_{2}$$

5 $${{{{{{\rm{CH}}}}}}}_{2}{{{{{\rm{ClX}}}}}}+{hv}\to {{{{{\rm{Cl}}}}}}+{{{{{\rm{X}}}}}}$$

6 $${{{{{\rm{X}}}}}}+{{{{{{\rm{O}}}}}}}_{3}\to {{{{{\rm{XO}}}}}}$$

7 $${{{{{{\rm{O}}}}}}}_{3}+{hv}\to {{{{{{\rm{O}}}}}}}^{1}{{{{{\rm{D}}}}}}+{{{{{{\rm{O}}}}}}}_{2}$$

8 $${{{{{{\rm{O}}}}}}}^{1}{{{{{\rm{D}}}}}}+{{{{{{\rm{H}}}}}}}_{2}{{{{{\rm{O}}}}}}\to 2{{{{{\rm{OH}}}}}}$$

9 $${{{{{\rm{Cl}}}}}}({{{{{\rm{Br}}}}}})+{{{{{\rm{VOC}}}}}}\to {{{{{{\rm{RO}}}}}}}_{2}+{{{{{\rm{HCl}}}}}}({{{{{\rm{HBr}}}}}})$$

10 $${{{{{{\rm{RO}}}}}}}_{2}+{{{{{\rm{NO}}}}}}\to {{{{{{\rm{NO}}}}}}}_{2}+{{{{{\rm{RO}}}}}}$$

11 $${{{{{\rm{RO}}}}}}+{{{{{{\rm{O}}}}}}}_{2}\to {{{{{{\rm{HO}}}}}}}_{2}+{{{{{\rm{products}}}}}}$$

12 $${{{{{{\rm{HO}}}}}}}_{2}+{{{{{\rm{NO}}}}}}\to {{{{{{\rm{NO}}}}}}}_{2}+{{{{{\rm{OH}}}}}}$$

13 $${{{{{\rm{XO}}}}}}+{{{{{{\rm{HO}}}}}}}_{2}\to {{{{{\rm{HOX}}}}}}$$

14 $${{{{{\rm{HOX}}}}}}+{hv}\to {{{{{\rm{X}}}}}}+{{{{{\rm{OH}}}}}}$$

Here, we conduct a set of long term (from the year 2000 to 2100 with an extra spin-up period of 40 years) emission-driven climate simulations utilizing the Community Earth System Model (CESM) (Methods; Supplementary Table 1) to evaluate the impact of RHS on CH_4_ loss rate, lifetime, burden, and radiative forcing during the 21st century. We adopt the free-running mode of CESM to facilitate the evolution of the climate system. Given the large range of CH_4_ levels projected under the different emission scenarios^26^, we explore here the middle- and high-end emission inventories of CH_4_ (instead of using surface lower-boundary conditions) to allow the free interaction of global CH_4_ with atmospheric chemistry, following the Representative Concentration Pathway (RCP) scenarios, RCP6.0 and RCP8.5, respectively. The variations of RHS sources considered in our study are actively linked with the physical, biogeochemical, and atmospheric changes to reflect the climate-chemistry feedback during the 21st century (Methods; ref. ^27^). In contrast to the well-established role of the chlorine atom in enhancing CH_4_ loss, our results show that halogens collectively decrease CH_4_ loss thereby increasing the CH_4_ lifetime. We show that the halogen-mediated increase in CH_4_ lifetime results in a significant enhancement in the present-day global CH_4_ burden and RF, which is predicted to continue increasing during the century.

## Results

### Burden of tropospheric reactive halogens in the 21st century

**Fig. 1:**
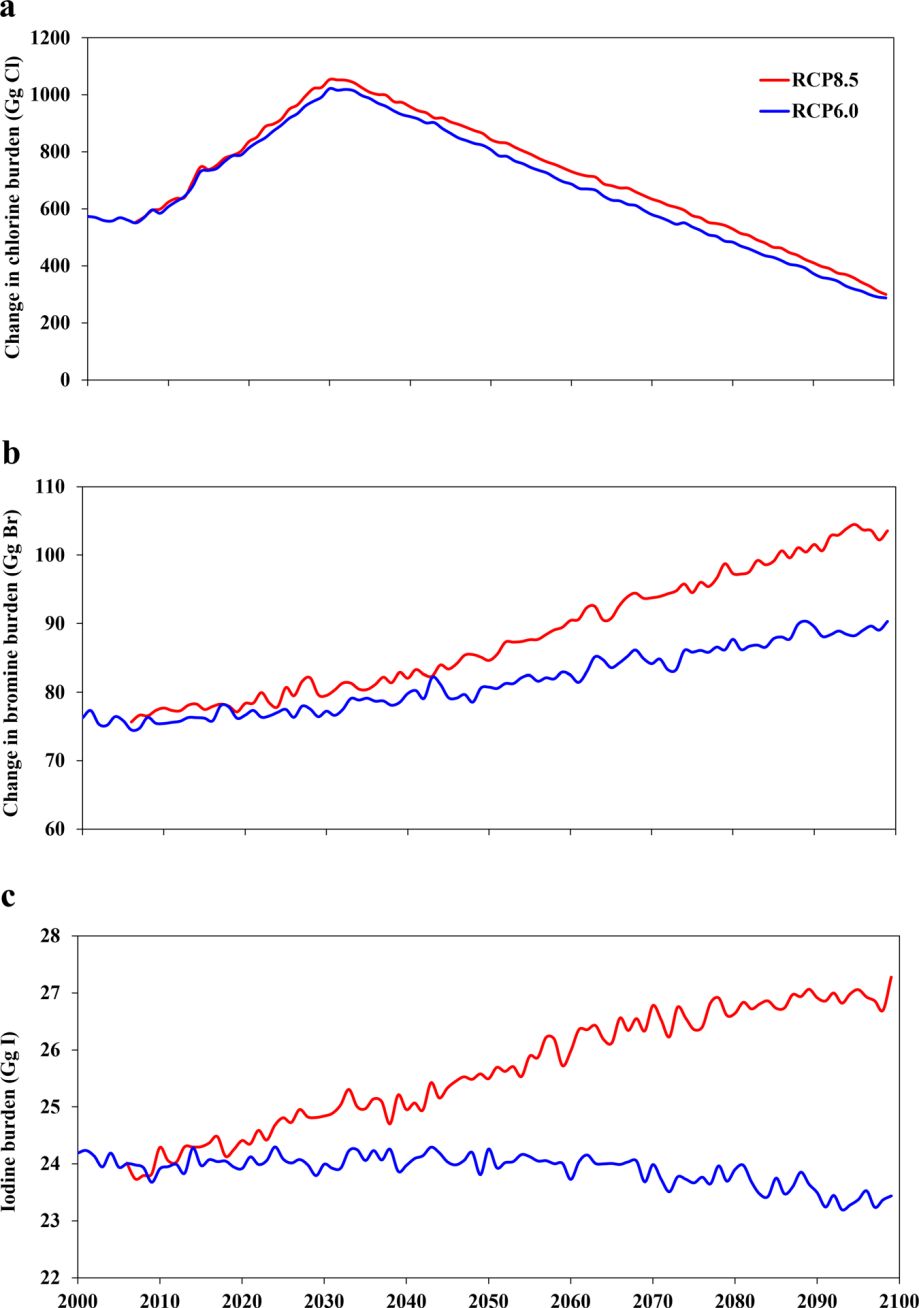
Reactive halogen species burden in the troposphere in the 21st century for RCP6.0 (blue line) and RCP8.5 (red line) scenarios. **a** reactive chlorine species (Cl_y_). **b** reactive bromine species (Br_y_). **c** reactive iodine species (I_y_). Definitions of Cl_y_, Br_y_, and I_y_ are included in Methods. The case without RHS (noHAL) contains a small amount of chlorine and bromine (but no iodine) species in the troposphere resulting from the breakdown of long-lived halogens in the stratosphere and the subsequent transport to the troposphere; therefore, this figure shows the RHS-mediated changes in tropospheric chlorine and bromine burdens but it shows the absolute levels of iodine species. Note that the results for RCP8.5 are only shown from 2006 to 2100 and the results before 2006 are identical to those in RCP6.0.

The inclusion of RHS sources and chemistry in CESM results in a large enhancement in active halogen levels in the atmosphere, particularly in the lower tropical troposphere (Fig. 1 and Supplementary Fig. 1). Note that in the simulation without RHS (noHAL), there is a small amount of chlorine and bromine species in the troposphere resulting from the breakdown of long-lived halocarbons (with a lifetime >180 days; e.g., CFCs and HCFCs) in the stratosphere and the subsequent transport to the troposphere; no iodine species are considered in the noHAL case (Methods). Thus Fig. 1 and Supplementary Fig. 1 depict the RHS-mediated changes in tropospheric chlorine and bromine but show the absolute levels of iodine species. Halogens, mainly iodine and bromine, chemical processing in the troposphere catalytically destroys O_3_ (Supplementary Fig. 2), whose photolysis is the main source of the OH radical (Eqs. 6–8). As a result, model runs with reactive halogens (HAL case) show global tropospheric OH is 5.8% (RCP6.0) and 5.2% (RCP8.5) lower relative to the noHAL reference runs (2000–2100 average; Supplementary Fig. 3). This suggests a significant indirect effect of halogens on CH_4_ through perturbing OH, thereby decreasing the CH_4_ chemical loss. On the other hand, RHS also supply Cl atoms which directly destroy CH_4_ (Eq. 2). By considering RHS (HAL), we estimate an increase of tropospheric Cl atoms by 175.7% in RCP6.0 and 232.7% in RCP8.5 averaged over the entire 21st century, compared to the case without RHS (noHAL) (Supplementary Fig. 4). These competing halogen-driven impacts highlight the importance of determining the trade-off between direct (Cl) and indirect (via OH) effects on CH_4_.

### Halogens impact on CH_4_ loss

**Fig. 2:**
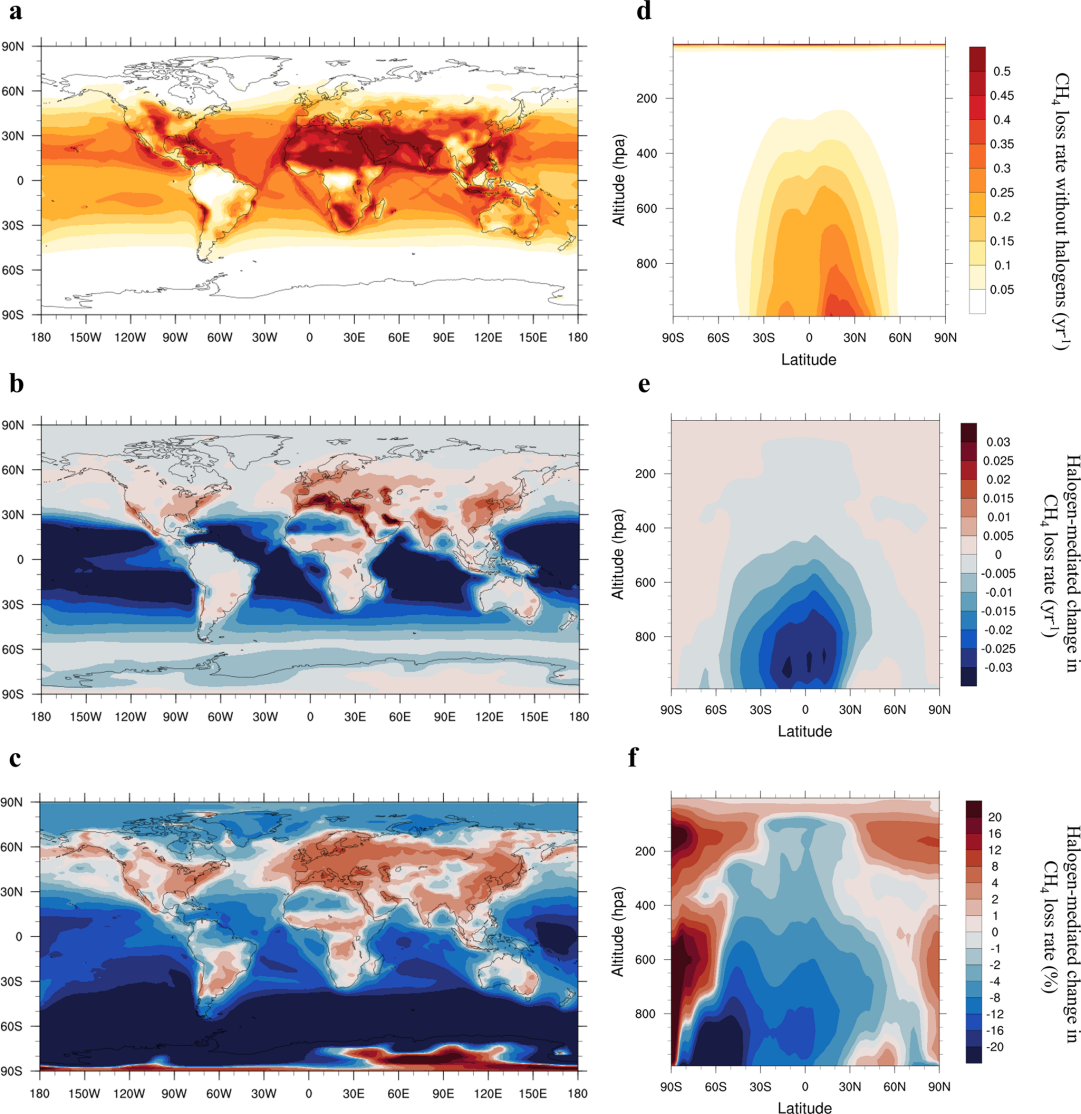
Spatial patterns of halogen-mediated change in CH_4_ loss rate averaged during the 21st century for RCP6.0 scenario. **a** Surface CH_4_ loss (yr^−1^) without halogens (noHAL). **b** Halogen-mediated changes in CH_4_ loss (yr^−1^) at the surface. **c** Same as (**b**) but in percentage. **d** Zonal distribution of CH_4_ loss (yr^−1^) without halogens (noHAL) showing the largest CH_4_ loss near-surface level in tropics. **e** Zonal distribution of halogen-mediated change in CH_4_ loss (yr^−1^). **f** Same as (**e**) but in percentage. Results for the RCP8.5 scenario are shown in Supplementary Fig. 5.

CH_4_ loss is mostly confined within the lower troposphere with the largest losses occurring near the surface in the tropics (Fig. 2 for RCP6.0 and Supplementary Fig. 5 for RCP8.5; Methods). The inclusion of halogens results in a marked hemispheric, regional, and vertical heterogeneity in CH_4_ loss. Most notably, halogens reduce the overall CH_4_ loss at the surface over the open ocean (i.e., clean environments) while increasing it over the continents (e.g., polluted regions). Considering halogen chemistry, the zonal mean tropospheric CH_4_ loss shows an intense reductionfrom the tropics to the south pole and from the surface to ~600 hPa, and an increase in the near-surface northern mid-latitudes and in the extratropical mid-to-upper troposphere (Fig. 2).

**Fig. 3:**
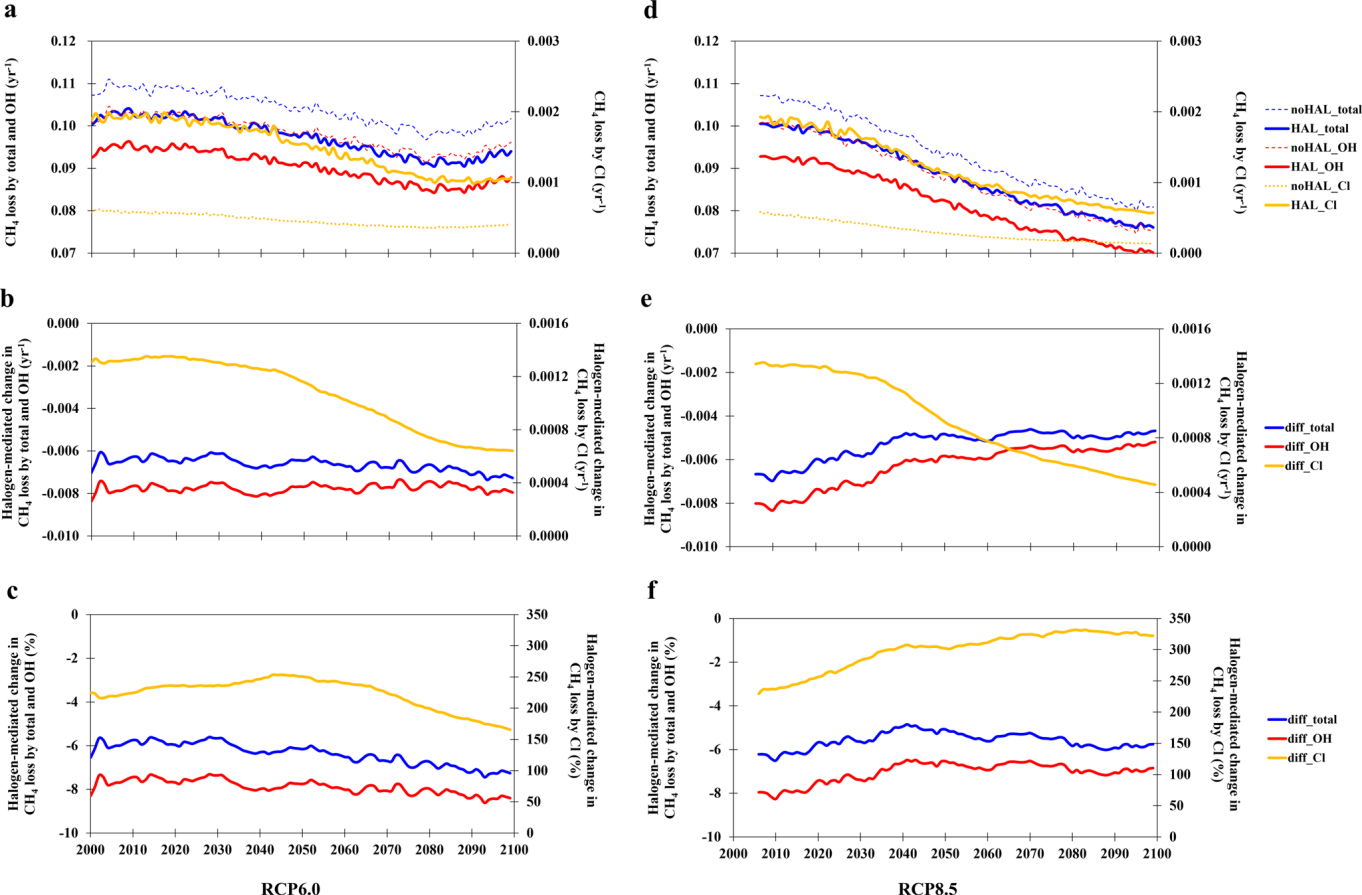
Evolution of halogen-mediated change in global CH_4_ loss rate during the 21st century for RCP6.0 (panels a, b, and c on the left) and RCP8.5 (panels d, e, and f on the right) scenarios. **a**, **d** The total CH_4_ chemical loss (yr^−1^; blue line) and the CH_4_ loss (yr^−1^) with respect to tropospheric OH (red line) and Cl (yellow line) radicals in both noHAL (dashed line) and HAL (solid line) cases. **b**, **e** Halogen-mediated change in CH_4_ loss in yr^−1^ for the total (blue), OH (red), and Cl (yellow) channels. **c**, **f** The same as (**b**, **e**) but in percentage. Note that the halogen-mediated changes are calculated with a moving average of 10 years (approximately the lifetime of CH_4_ in the atmosphere). The halogen-mediated changes in CH_4_ loss rates via total channels and the OH channel are negative (left *Y*-axis on panels **b**, **c**, **e**, **f**) while those via the Cl channel are positive (right *Y*-axis).

Figure 3 shows the time series of annual global CH_4_ loss and its changes due to reactive halogens during the 21st century for both RCP scenarios. Regardless of including or neglecting RHS in the model, CH_4_ loss shows a consistent decreasing trend for RCP6.0 and RCP8.5 in the 21st century. This is associated with the decreasing trend of the tropospheric OH abundance (Supplementary Fig. 6), as a result of increasing trend of CH_4_ emissions in the 21st century^26^. The consideration of RHS in the CESM model, in active connection with the climate system, leads to a significant reduction in tropospheric O_3_ (Supplementary Fig. 2) and therefore OH (Supplementary Fig. 3), mostly due to iodine and bromine (ref. ^27,28^), but a large enhancement in Cl atom levels (Supplementary Fig. 4) averaged over the entire 21st century. Although the tropospheric OH burden is reduced by RHS, the contribution of the dominant OH-driven pathway to the total CH_4_ loss in all simulations is one to two orders of magnitude larger than that of the Cl-driven channel (Fig. 3a). The consideration of halogens leads to significant changes in both CH_4_ loss channels (Fig. 3b, c). The enhancement of Cl-driven CH_4_ loss is only a fraction of the large reduction in OH-driven CH_4_ loss due to the combined halogens, thereby resulting in a net reduction of the total CH_4_ loss (Fig. 3b, c).

### Halogens impact on CH_4_ lifetime

**Fig. 4:**
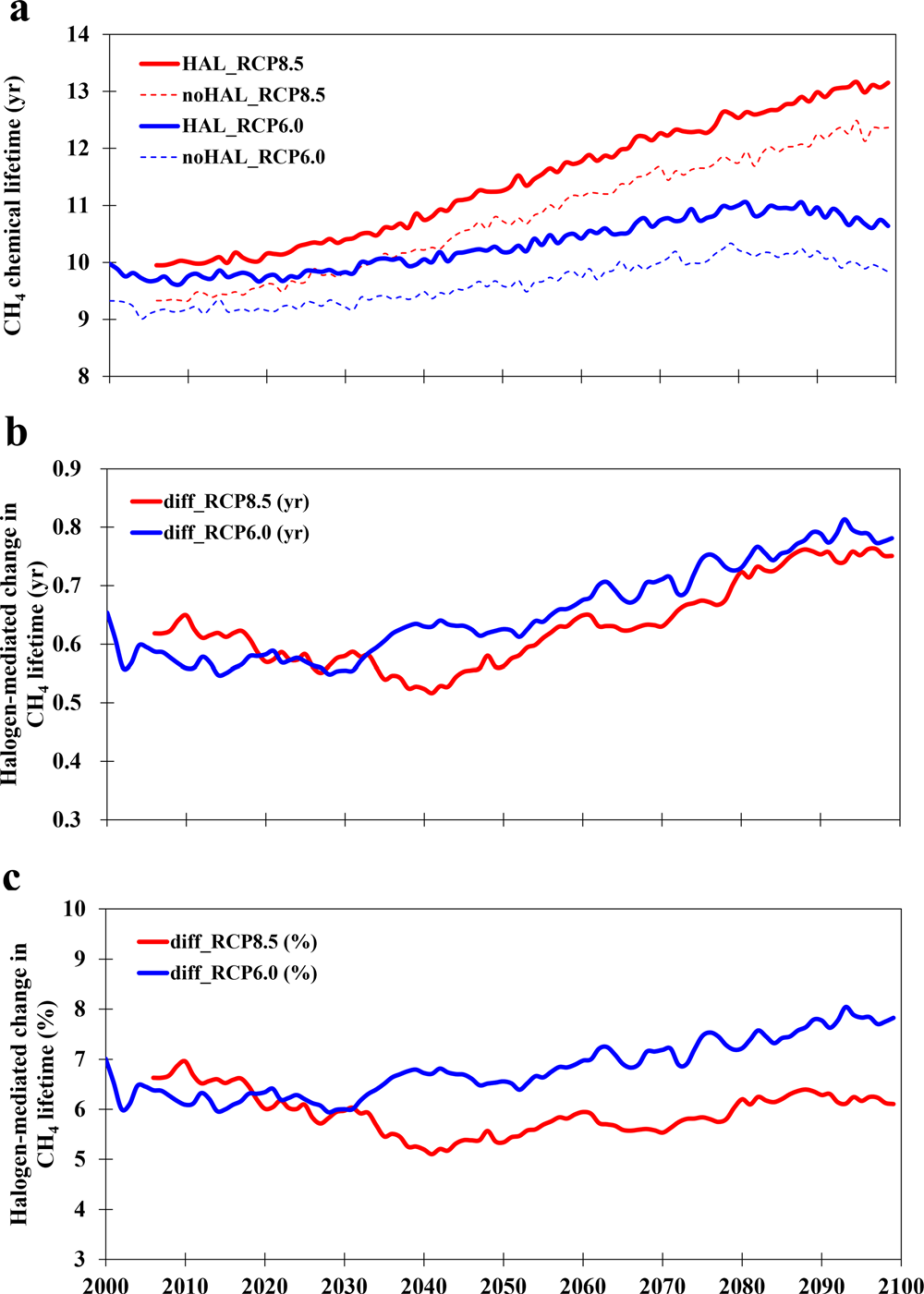
Evolution of halogen-mediated change in CH_4_ chemical lifetime in the 21st century for RCP6.0 (blue line) and RCP8.5 (red line) scenarios. **a** Global CH_4_ chemical lifetime (yr) for simulations with (HAL, solid line) and without (noHAL, dashed line) halogens; **b** Halogen-mediated change in CH_4_ lifetime in absolute term (yr); **c** The same as (**b**) but in percentage. Note that the halogen-mediated changes are calculated with a moving average of 10 years (approximately the lifetime of CH_4_ in the atmosphere). The maximum halogen-mediated effect occurs in 2093 for RCP6.0 with an enhancement of ∼0.9 yr (9.4%). Note that the results for RCP8.5 are only shown from 2006 to 2100 and the results before 2006 are identical to those in RCP6.0. CH_4_ lifetime with respect to tropospheric OH is shown in Supplementary Fig. 7.

The halogen-driven reduction of CH_4_ loss increases the lifetime of CH_4_ in the atmosphere. Our simulations show that the total CH_4_ lifetime in all cases presents an increasing trend, from ~9.5 yr at present-day (2000**–**2019) to ~10.5 yr (RCP6.0) or ~12.5 yr (RCP8.5) by the end of the century (2080–2099) (Fig. 4; Methods). Halogens enhance the total CH_4_ lifetime by ~0.6 yr (~6.3%) at present and by ~0.8 yr (8.0%) in the RCP6.0 scenario or ~0.7 yr (6.1%) in RCP8.5 by 2100.

We also find that halogens increase the CH_4_ lifetime with respect to (w.r.t.) OH during the whole 21st century (Supplementary Fig. 7). For present-day conditions (averaged between 2000 and 2019), the CH_4_ lifetime w.r.t. OH is increased from 9.8 to 10.6 yr (or 8.2%) for the RCP6.0 scenario and from 10.0 to 10.8 yr (or 8.4%) for RCP8.5. A previous chemical transport model study estimated a similar increase (10.8%) in CH_4_ lifetime (w.r.t. OH) due to RHS using a 2-year simulation for present-day conditions (2004–2005)^21^. The current study shows that reactive halogen chemistry in the troposphere is predicted to consistently decrease the global tropospheric OH levels throughout the 21st century (Supplementary Fig. 6), which, in turn, reduces the CH_4_ loss rate towards OH (Fig. 3), thereby increasing the CH_4_ lifetime w.r.t. OH (Supplementary Fig. 7). It is noteworthy that previous modeling studies reported CH_4_ lifetimes w.r.t. OH of 9.3 (±0.9) yr (ref. ^10^) and 8.7 (±1.1) yr (ref. ^13^) which are lower than the observational estimate of 11.2 (±1.3) yr (ref. ^11^). The results with and without RHS suggest that the inclusion of RHS increases the CH_4_ lifetime w.r.t. OH from ≤10 yr to >10.5 yr, which is closer to the observation-based estimate and therefore helps to reduce the gap between modeled CH_4_ lifetimes and observational estimates.

### Halogens impact on CH_4_ burden

**Fig. 5:**
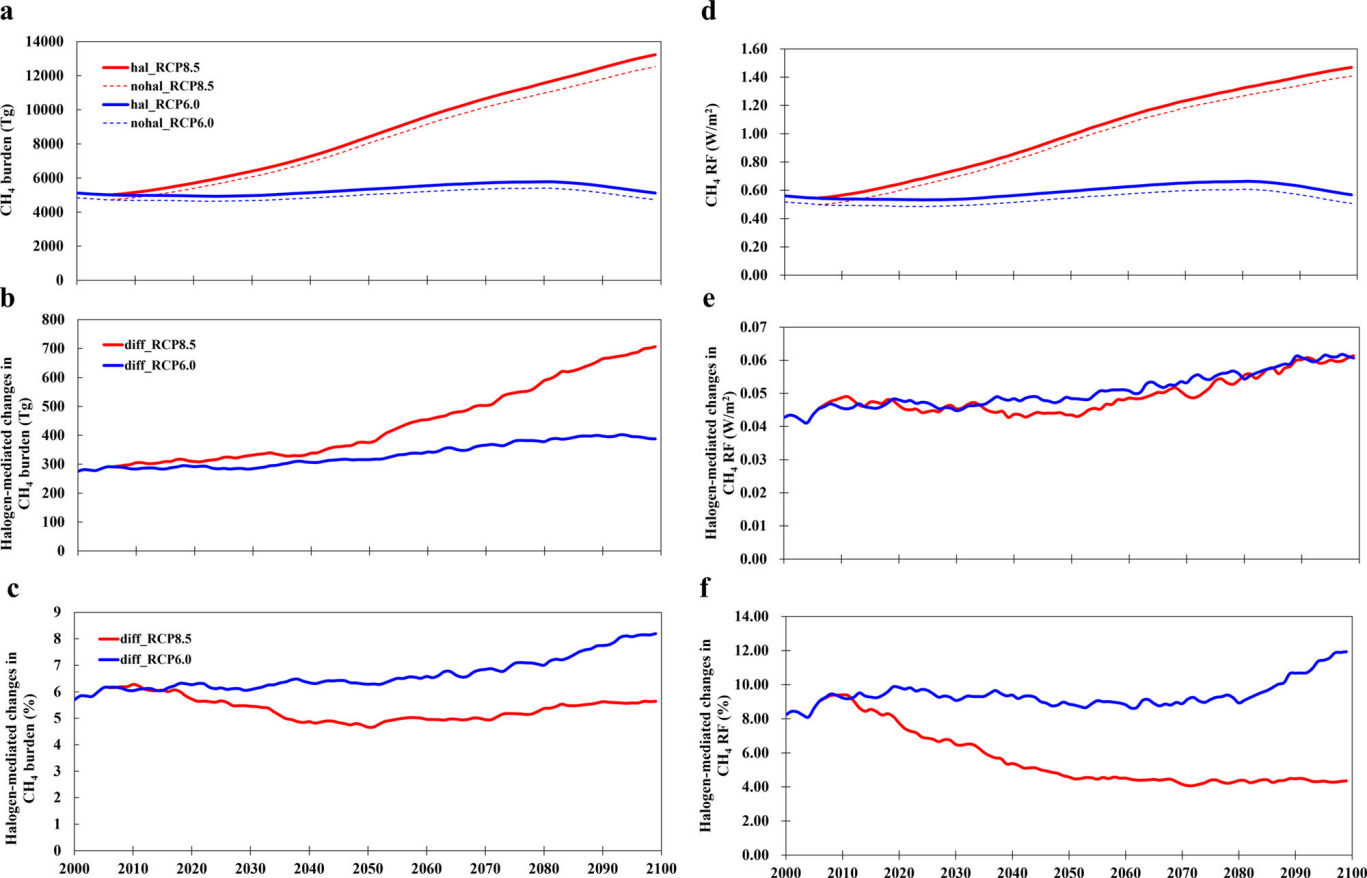
Evolution of halogen-mediated change in CH_4_ burden and radiative forcing in the 21st century for RCP6.0 (blue line) and RCP8.5 (red line) scenarios. **a** CH_4_ burden (Tg) for simulations with (HAL, solid line) and without (noHAL, dashed line) halogens; **b** Halogen-mediated change in CH_4_ burden in the unit of Tg; **c** The same as (**b**) but in percentage; **d** Radiative forcing (W/m^2^) of CH_4_ compared to pre-industrial level with (HAL, solid line) and without (noHAL, dashed line) halogens; **e** Halogen-mediated change in CH_4_ radiative forcing in the unit of W/m^2^; **f** The same as (**e**) but in percentage. Note that the results for RCP8.5 are only shown from 2006 to 2100 and the results before 2006 are identical to those in RCP6.0.

Halogen-mediated chemical processing of CH_4_, both direct and indirect, significantly impacts the global atmospheric CH_4_ burden (Fig. 5a–c; Supplementary Table 2). The CH_4_ atmospheric burden in the RCP6.0 scenario varies around 5000 Tg along the 21st century while that in the RCP8.5 dramatically rises to ~13000 Tg by the end of the century. Our results show that halogens increase the global CH_4_ burden by ~300 Tg in the 2000s and ~400 Tg (RCP6.0) or ~700 Tg (RCP8.5) by the end of the 21st century (Fig. 5b). In relative terms, CH_4_ burdens are increased due to halogens by ~6% in the 2000s and ~8% in RCP6.0 or ~5% in RCP8.5 by 2100. The CH_4_ burden change (in %) in RCP6.0 remains nearly unchanged from the early- to mid-century, and increases towards the end of the century; while in the RCP8.5 scenario, it shows a slightly decreasing trend in the first half of the century and a resumed increasing trend in the second half of the century (Fig. 5c). During the past few decades, observations reported an annual increase of 13 Tg/yr (over the 2008–2017 period), 3 to 8 Tg/yr (2000–2009), 12 Tg/yr (1990–1999), and 30 Tg/yr (1980–1989) in atmospheric CH_4_ burden^4,5^, suggesting a global total increase of 280–330 Tg in the last three decades and 580–630 Tg in the last four decades. This is comparable to our calculated halogen-mediated change in CH_4_ burden of 300 Tg in 2020 and 400 Tg (RCP6.0) to 700 Tg (RCP8.5) by 2100. The present study is the first report of the RHS-induced change in atmospheric CH_4_ burden, and indicates that the impact of neglecting halogens in model predictions of the atmospheric CH_4_ burden is of equivalent magnitude to the omission of its observed global increase during the last three to four decades.

### Halogens impact on CH_4_ RF

RF is a critical parameter adopted by the climate science community to quantify the strength of various factors (e.g., GHG) in regulating the radiation budget of the Earth and to compare the contribution of these factors to global warming and climate change^12^. The present study calculates the halogen-mediated CH_4_ RF, compared to the pre-industrial period, and its variations over the 21st century by applying the latest methodology reported in ref. ^29^ (Methods).

The global CH_4_ RF in the 21st century for all model simulations (Fig. 5d) follow the same trend as the CH_4_ atmospheric burden (Fig. 5a): CH_4_ RF in the RCP6.0 scenario remains at ~0.6 W/m^2^ during the century while that in RCP8.5 drastically increases to ~1.5 W/m^2^ by 2100. The global CH_4_ RF change induced by halogens for the two RCP scenarios is similar in terms of the absolute values (W/m^2^; Fig. 5e), but shows a comparatively larger percentage change in RCP6.0 (Fig. 5f). Halogen-induced CH_4_ RF increases by 0.04 W/m^2^ (8%) in the early 2000s and 0.06 W/m^2^ (12% in RCP6.0 and 4.5% in RCP8.5) towards 2100, thereby enhancing global CH_4_ induced warming in the 21st century. Note that the pre-industrial to present-day RF increases due to the key well-mixed GHGs are 1.95 (CO_2_), 0.62 (CH_4_), and 0.18 (N_2_O) W/m^2^ (ref. ^29^). Thus, the halogen effect on RF via changing CH_4_ abundances, currently not considered in climate models, is estimated to represent up to one-third of the historical N_2_O RF and ~10% of the CH_4_ RF.

### Chlorine impact on CH_4_

Traditionally, the halogen-mediated impact on CH_4_ refers to the direct effect of the Cl atom in increasing CH_4_ loss^4^. Here, we conduct a sensitivity simulation with only chlorine sources and chemistry but without bromine or iodine (OnlyCl case; Supplementary Table 1). Our results show that chlorine leads to a 1.9% decrease in tropospheric O_3_ averaged in the 21st century (and 2.7% from 2000 to 2019), while the halogens in total cause a 14.6% ozone reduction averaged in the 21st century (and 15.6% from 2000 to 2019). The estimated reduction in tropospheric OH burden due to chlorine (2.5% for present-day and 2.4% for 21st century) and combined halogens (5.9% for present-day and 5.8% for 21st century) are within the range of previous reports^21,22^.

Here, we further diagnose the OH budget and its changes with the presence of only chlorine and all halogens (Supplementary Fig. 8). The production of OH (Tg yr^−1^; averaged in the 21st century) is decreased by 1.3% due to chlorine and by 7.7% due to the three halogens combined; such decrease in OH production is mainly driven by O_3_ loss. The loss of OH (yr^−1^; normalized by its burden and averaged in the 21st century) is increased by 1.9% due to chlorine and by 1.0% due to halogens; such increase in OH loss is due to the larger levels of CH_4_, CO, and VOCs resulting respectively from the chlorine- and halogen-induced lower OH production.

**Fig. 6:**
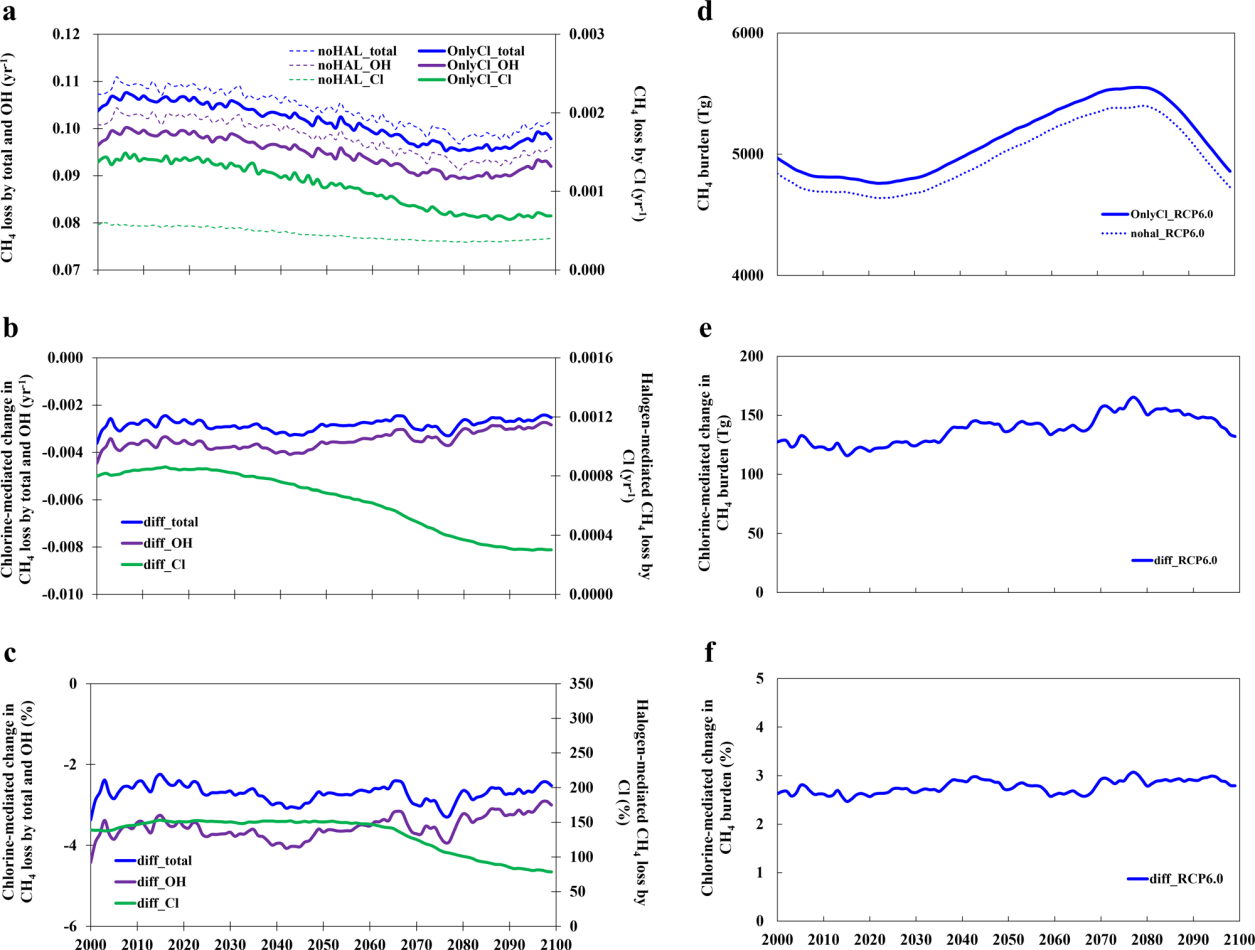
Evolution of the chlorine-mediated change in CH_4_ loss rate and burden in the 21st century in the OnlyCl case. **a** The total CH_4_ loss (yr^−1^; blue line) and the CH_4_ loss (yr^−1^) by OH (purple line) and Cl (green line) radicals in both noHAL and OnlyCl cases. **b** Chlorine-mediated change in CH_4_ loss in yr^−1^ for the total (blue), OH (purple line), and Cl (green line) channels. **c** The same as (**b**) but in percentage. Note that the chlorine-mediated changes are calculated with a moving average of 10 years (approximately the lifetime of CH_4_ in the atmosphere). **d** CH_4_ burden (Tg) in OnlyCl (solid line) and noHAL (dashed line) cases; **e** Chlorine-mediated change in CH_4_ burden in units of Tg; **f** The same as (**e**) but in percentage.

The reduced global OH burden due to chlorine chemistry, via reducing OH production and increasing its loss, particularly in the vast open ocean and free troposphere, indirectly reduces CH_4_ loss and enhances its burden (Fig. 6). Hence, our results show that even when the other two halogens (Br and I) are not considered, the net effect of chlorine is to reduce the global CH_4_ loss. The Cl-driven increase in the CH_4_ burden (Fig. 6e) is about 40% of that of combined halogens at present and decreases to 30% by the end of the century due to the future overall enhancement in the indirect contribution of bromine and, mostly, iodine to the CH_4_ loss (Fig. 1).

## Discussion

The direct emitting sources and soil sinks of CH_4_ have been the focus of intense research. A few recent studies reported that the future evolution of natural sources and sinks of CH_4_ is subject to many environmental factors^30–33^. In the present study, we neglect such variations in natural CH_4_ sources. Instead, we adopt both the mid- and high-end anthropogenic CH_4_ emission scenarios and keep the natural variation constant in the 21st century (Supplementary Table 2). With it, we aim at properly capturing the potential variation of future CH_4_ burden and addressing the opposite contribution from the direct (Cl-driven) and indirect (via OH) effect of RHS on CH_4_ chemical losses. Thus, our study provides a probable range of the RHS effect on CH_4_ (loss rate, lifetime, burden, and radiative forcing) under two significantly different climate scenarios in the 21st century. The results show that even though the CH_4_ emission doubles in RCP8.5 compared to RCP6.0, the reactive halogen impacts on CH_4_ are similar between the two scenarios.

The dominant loss of atmospheric CH_4_ has been consistently reported to be its chemical reactions with atmospheric oxidants (OH and Cl)^4,5^. Yet, previous CH_4_ modeling studies mostly use lower-boundary conditions of CH_4_, i.e., resetting the surface CH_4_ mixing ratios at each time step to its predefined latitude-dependent values, which largely neglects the chemical processing of atmospheric CH_4_. Our study, on the other hand, applies direct CH_4_ emissions from spatially-resolved inventories of anthropogenic and natural sources, which facilitates a more realistic representation of atmospheric CH_4_ abundances via its chemical processing and climate feedbacks (Fig. 2). Specifically, changes in atmospheric oxidants due to RHS interact with atmospheric CH_4_ in a globally consistent manner, allowing a complete chemistry-climate coupling. We recommend that future climate simulations should apply emission inventories for CH_4_ and other short-lived climate forcers to fully represent their complex and non-linear connections with the components of the climate system.

When applying the emission inventories of CH_4_ in Earth system modeling, one needs to carefully consider the spin-up time so that the atmospheric CH_4_ burden and RF are stabilized. We have conducted several test cases with different spin-up periods (10, 20, 30, and 40 years; Supplementary Table 3). We find that a spin-up time of 40 years, approximately four times the atmospheric lifetime of CH_4_, is required to provide a stabilized CH_4_ burden, as indicated by the very minor difference (<2%) of CH_4_ burden in the year 2000 between the 30- and 40-years spin-up cases. Therefore, we recommend that the simulations of CH_4_ burden and RF should be conducted with sufficient spin-up to reflect the non-linear connection between atmospheric chemistry and CH_4_.

The sources of RHS in the future are expected to vary in accordance with the future climate system and anthropogenic activity. The present study forecasts the natural (mostly oceanic) sources of RHS online depending on the various parameters simulated in our Earth system model (e.g., net primary production, wind speed, sea-surface temperature, air pollutants levels, etc.) following our previous study on the evolution of RHS during the 21st century^27^. In addition, here we include continental halogen sources and estimate the range of the anthropogenic and biomass burning emissions of RHS based on the emissions of other anthropogenic air pollutants (Methods). In such a manner, we are able to provide a range of the future variation of RHS, both over the open ocean and over terrestrial domains.

Although the observational evidence of RHS in the atmosphere has been growing in the past two decades, more future field measurements and laboratory experiments are desired to further increase our understanding of the sources, sinks, and chemical fate of RHS, and multiscale modeling simulations are required to evaluate the holistic effects of RHS on oxidants and short-lived climate forcers like CH_4_.

The full impact of RHS on the chemical loss of CH_4_ and its representation in chemistry-climate models has attracted little attention to this date. The existing climate assessment reports (e.g., ref. ^12^) and CH_4_ studies (e.g., ref. ^4^) only consider the direct effect of RHS on CH_4_ (via Cl) but exclude the indirect effect (via OH); in doing so, these studies implied that the presence of reactive halogens leads to more CH_4_ removal. However, as highlighted above, our study found that the indirect effect of RHS (via OH) dominates the overall RHS impact on CH_4_ meaning that the consideration of RHS in the climate system results in less CH_4_ loss.

The benchmark chemical schemes considered in the ongoing Phase 6 of the Coupled Model Intercomparison Project (CMIP6) project^34^ do not include the reactive halogen sources and chemistry discussed in the present work. By neglecting the full impact of tropospheric reactive halogens in CH_4_ and climate studies, one is omitting a substantial impact on the atmosphere’s oxidizing potential, as well as direct and indirect chemical effects of halogens on CH_4_ and their variations in the 21st century.

This study attempts to bridge this gap in our knowledge with 100-year climate model simulations. Our results show that the inclusion of RHS in CESM helps to reduce the existing gap between modeled and observationally-derived CH_4_ lifetime. We estimate that the RHS impact on the global CH_4_ burden is equivalent in magnitude to the global CH_4_ burden increase observed in the last three to four decades, and that the RHS-induced CH_4_ RF change is up to 1/3 of the third-largest well-mixed GHG (N_2_O). We also find that the RHS effects on CH_4_ parameters (loss rate, lifetime, burden, and RF) are not constant but rather variable under the changing climate in the 21st century, which is associated with the projected trend in CH_4_ emission^26^ as well as the online simulated future evolution of RHS burdens (Fig. 1). In light of these results, we recommend that reactive halogen chemistry in the troposphere should be incorporated into climate model assessments of CH_4_.

## Methods

### CH_4_ sources and sinks

The sources of atmospheric CH_4_ include direct emission and chemical production. The emitting sources consist of anthropogenic sources (including the agricultural sector, landfill, fossil fuel industry, and biomass/biofuel burning) and natural ones (mostly from wetlands). Supplementary Table 2 summarizes the CH_4_ emission strength of each source used in the present study, at present time, in the future, and averaged during the 21st century for both RCP6.0 and RCP8.5 scenarios. Anthropogenic sources in the past few decades have been reported to be in the range of 319 (255–357) to 380 (359–407) Tg/yr using top-down and bottom-up methods; while the natural sources have a similar emission between 218 (179–273) and 386 (259–532) Tg/yr (refs. ^4,5,35^). Our model includes the atmospheric oxidation of C_3_H_6_ (annual emission of ~5Tg/yr) which produces CH_4_ with a production yield of 0.08 (ref. ^36^). Such a small chemical production of CH_4_ (<1 Tg/yr) is negligible compared to the direct CH_4_ sources (>500 Tg/yr).

The sinks of CH_4_ include chemical loss and soil sink (dry deposition). There are three chemical loss pathways of CH_4_ in the atmosphere, mainly OH (Eq. 1) and Cl (Eq. 2) and also a negligible channel via O(^1^D) (mostly from the photolysis of O_3_). The chemical loss of CH_4_ has been reported to be 505 (459–516) to 604 (483–738) Tg/yr in the past few decades; the soil sink contributes between 28 (9–47) to 40 (37–47) Tg/yr (refs. ^4,5,35^).

### Calculation of CH_4_ loss, lifetime, burden, and radiative forcing

The spatial distribution of CH_4_ loss rate (yr^−1^; e.g., Fig. 2) is the CH_4_ chemical loss rate in each location (in molecules/cm^3^/s; via reactions with OH, Cl, and O^1^D) normalized by the CH_4_ concentration (molecules/cm^3^) and transformed to be in units of yr^−1^.

The global integrated CH_4_ chemical loss (yr^−1^; e.g., Fig. 3) is the globally integrated loss rate (Tg/yr) normalized by the global burden (Tg). The CH_4_ loss due to OH (yr^−1^; e.g., Fig. 3) and that due to Cl (yr^−1^) are the global CH_4_ loss (Tg/yr) arising only from the reactions with OH and Cl, respectively, normalized by the global CH_4_ burden (Tg).

The total CH_4_ chemical lifetime (yr; e.g., Fig. 4) is the inverse of the global integrated CH_4_ chemical loss (yr^−1^). The CH_4_ lifetime with respect to OH (yr) is the inverse of the global integrated CH_4_ loss due to OH (yr^−1^).

The global CH_4_ burden (Tg; e.g., Fig. 5) is the sum of CH_4_ mass (molecules) in each grid at each vertical level and is transformed to be in the unit of Tg.

We follow the radiative forcing equation (Eq. 15) proposed in ref. ^29^ to calculate the radiative forcing of CH_4_ (W/m^2^; e.g., Fig. 5).

15 $${RF}({{CH}}_{4})=\, 	(-1.3\;\times {10}^{-6}\times (M+{M}_{0})/2-8.2\times {10}^{-6} \\ 	\times (N+{N}_{0})/2+0.043) \times \left(\sqrt{M}-\sqrt{{M}_{0}}\right)$$

In this equation, M is the global average mixing ratio of CH_4_ in ppbv at the time of interest, while M_0_ is that at the initial time. Similarly, N and N_0_ are those of N_2_O. The values of M and N are extracted from the CESM simulation, and M_0_ and N_0_ are set as 722 ppbv for CH_4_ and 270 ppbv for N_2_O, respectively, for the pre-industrial level (the year 1750; ref. ^29^).

Note that replacing Eq. 15 with the radiative forcing expression used in IPCC^12^ results in the same conclusion (up to 0.06 W/m^2^ enhancement in CH_4_ radiative forcing due to halogens by the end of the century).

### Reactive halogen chemistry

Reactive halogen species consist of organic and inorganic chlorine (including Cl, ClO, Cl_2_, Cl_2_O_2_, OClO, COCl_2_, HOCl, ClONO_2_, HCl, BrCl, ClNO_2_, ICl, CH_2_Cl_2_, C_2_Cl_4_, CH_2_BrCl, CH_2_ICl, CHBr_2_Cl, CHBrCl_2_, CHCl_3_, C_2_H_4_Cl_2_, and C_2_HCl_3_), bromine (including Br, BrO, HOBr, BrONO_2_, HBr, BrCl, Br_2_, BrNO_2_, IBr, CHBr_3_, CH_2_Br_2_, CH_2_BrCl, CHBr_2_Cl, CHBrCl_2_, CH_2_IBr), and iodine species (I, I_2_, IO, OIO, HI, HOI, INO, INO_2_, IONO_2_, IBr, ICl, I_2_O_2_, I_2_O_3_, I_2_O_4_, CH_2_I_2_, CH_2_IBr, CH_2_ICl, CH_3_I) which have a lifetime lower than 180 days. The total chlorine, bromine, and iodine abundance (Cl_y_, Br_y_, I_y_, respectively) accounts for the sum of the individual halogen species multiplied by their Cl, Br, and I atomicity (Fig. 1 and Supplementary Fig. 1).

As discussed in the main text, the reduction effect of RHS on OH in a clean environment decreases CH_4_ loss and the enhancement effect of RHS on OH in the polluted area increases CH_4_ loss. In addition, the presence of Cl atoms directly increases CH_4_ loss through Eq. 2. Thus, the overall effect of RHS on CH_4_ loss (and therefore lifetime, burden, and radiative forcing) depends on the efficiency of the above reactions in different regions which requires a full assessment with global models coupled with comprehensive halogen sources and chemistry.

The heterogeneous reactions of halogen species on sea-salt aerosol (SSA) and other tropospheric aerosols, which are simulated online in the CESM model depending on the surface wind and anthropogenic emissions, act as sources or recycling processes of reactive halogens. The implementations of halogen release from the sea-salt aerosol in the CESM model, e.g., via HOBr/HOI heterogeneous uptake (Eqs. 16–24), have been described in detail in previous works, e.g., refs. ^37,38^. Note that these non-stoichiometric reactions result in a net source of reactive chlorine and bromine to the atmosphere but only a change in gas-phase partitioning for iodine. In the current study, we added the N_2_O_5_ and HNO_3_ induced chlorine release pathway from the sea-salt aerosol (Eqs. 25–26) and recycling of chlorine process on other tropospheric aerosols (Eq. 27) following ref. ^7^.

16 $${{{{{\rm{HOCl}}}}}}+{{{{{\rm{SSA}}}}}}\to {{{{{{\rm{Cl}}}}}}}_{2}$$

17 $${{{{{{\rm{ClONO}}}}}}}_{2}+{{{{{\rm{SSA}}}}}}\to {{{{{{\rm{Cl}}}}}}}_{2}$$

18 $${{{{{{\rm{ClNO}}}}}}}_{2}+{{{{{\rm{SSA}}}}}}\to {{{{{{\rm{Cl}}}}}}}_{2}$$

19 $${{{{{\rm{HOBr}}}}}}+{{{{{\rm{SSA}}}}}}\to 0.65{{{{{{\rm{Br}}}}}}}_{2}+0.35{{{{{\rm{BrCl}}}}}}$$

20 $${{{{{{\rm{BrONO}}}}}}}_{2}+{{{{{\rm{SSA}}}}}}\to 0.65{{{{{{\rm{Br}}}}}}}_{2}+0.35{{{{{\rm{BrCl}}}}}}$$

21 $${{{{{{\rm{BrNO}}}}}}}_{2}+{{{{{\rm{SSA}}}}}}\to 0.65{{{{{{\rm{Br}}}}}}}_{2}+0.35{{{{{\rm{BrCl}}}}}}$$

22 $${{{{{\rm{HOI}}}}}}+{{{{{\rm{SSA}}}}}}\to 0.5{{{{{\rm{IBr}}}}}}+0.5{{{{{\rm{ICl}}}}}}$$

23 $${{{{{{\rm{IONO}}}}}}}_{2}+{{{{{\rm{SSA}}}}}}\to 0.5{{{{{\rm{IBr}}}}}}+0.5{{{{{\rm{ICl}}}}}}$$

24 $${{{{{{\rm{INO}}}}}}}_{2}+{{{{{\rm{SSA}}}}}}\to 0.5{{{{{\rm{IBr}}}}}}+0.5{{{{{\rm{ICl}}}}}}$$

25 $${{{{{{\rm{N}}}}}}}_{2}{{{{{{\rm{O}}}}}}}_{5}+{{{{{\rm{SSA}}}}}}\to {{{{{{\rm{ClNO}}}}}}}_{2}+{{{{{{\rm{HNO}}}}}}}_{3}$$

26 $${{{{{{\rm{HNO}}}}}}}_{3}+{{{{{\rm{SSA}}}}}}\to {{{{{\rm{HCl}}}}}}$$

27 $${{{{{{\rm{N}}}}}}}_{2}{{{{{{\rm{O}}}}}}}_{5}+{{{{{\rm{HCl}}}}}}\to {{{{{{\rm{ClNO}}}}}}}_{2}+{{{{{{\rm{HNO}}}}}}}_{3}$$

### CESM model setup

In the present study, we utilize a well-validated earth system model, CESM^39^, with an active atmospheric chemistry component (CAM-Chem^36^) to quantify the RHS effect on CH_4_. We conducted four main simulations with and without the direct sources of reactive halogens (species with a lifetime <6 months; refer to the “Reactive halogen chemistry” section for the species list) for both RCP6.0 and RCP8.5 scenarios, including noHAL_6.0, HAL_6.0, noHAL_8.5, and HAL_8.5 (Supplementary Table 1). Note that in the noHAL case, the long-lived halogen species (those with a lifetime >6 months; e.g., halons, CFC, etc.) are included to maintain a realistic stratosphere and some of the oxidation products of these long-lived halogens in the stratosphere will re-enter the troposphere following stratospheric intrusion/subsidence. The noHAL case is the standard setup used in previous chemistry-climate model projections of CH_4_. The changes in atmospheric composition (CH_4_, halogens, O_3_, OH, etc.) between HAL and noHAL represent the effect of RHS. An additional CESM sensitivity test with only chlorine sources and chemistry is also conducted (Supplementary Table 1) to isolate the chlorine effect on CH_4_.

All simulations started in 1960 and were run for 140 years with the first 40 years (1960–1999) discarded as spin-up results and the remaining 100 years results (2000–2099) were used for analysis in the present work. Note that 40 years of spin-up are required to ensure the atmospheric CH_4_ burden stabilization. The present time is defined as the mean between the years 2000 to 2019, and the end of the century is 2080 to 2099.

The emissions of air pollutants follow previous RCP studies^40^ based on the model configuration of ref. ^27^. Note that most models use CH_4_ as a lower-boundary condition (LBC), specified from estimations using MAGICC^41^ or similar models. However, here we use the emission inventory of anthropogenic and biomass burning sources for both RCP scenarios instead of the homogeneous surface LBC, because utilizing LBC is not able to fully represent the distribution, transport, and chemistry of CH_4_ in the atmosphere nor the spatial variation at the surface layer. For the natural source of CH_4_, we apply the wetland emission inventory compiled by ref. ^42^ for the period 2009 to 2010 and we apply their highest scenario (207.5 Tg/yr; to account for the other natural sources not considered here) as a cyclical emission throughout the simulation period. The average emission intensities of the three CH_4_ sources at the present time, at the end of the century, and during the entire 21st century are summarized in Supplementary Table 2.

Natural sources of RHS have been documented in detail in ref. ^27^. Briefly, sources of natural halogens, released primarily from the ocean, include biogenic and abiotic routes. Biogenic VSLS (e.g., CHBr_3_, CH_2_Br_2_, CH_3_I, and CH_2_ICl) are the result of marine organisms’ metabolism, such as phytoplankton and macro-/microalgae. Present-day fluxes of naturally emitted biogenic VSLS follow the ref. ^43^ emission inventory. Climate-induced changes, including physical (sea-surface temperatures) and biogeochemical (marine primary production) factors, drive changes in natural halocarbon fluxes^27^. Iodine species (HOI and I_2_) are directly emitted from the ocean following an abiotic route^44^, and their online fluxes are governed by surface ozone concentrations, wind speed, and sea-surface temperatures, which are computed online in CESM^45^. Four losses of HCl are considered in the CESM model, including chemical loss, removal by ice-uptake, in-cloud washout, and dry deposition.

In the present work, we also include anthropogenic and biomass burning sources of organic and inorganic chlorine species (with a lifetime <180 days). For organic chlorine, we adopt the emission inventory of the two dominant species (CH_2_Cl_2_ and C_2_Cl_4_) proposed by ref. ^46^ and the LBC of other anthropogenic organic chlorine species (CHCl_3_, C_2_H_4_Cl_2_, and C_2_HCl_3_) reported in ref. ^7^ for the present time (the early 2000s to mid-2010s). We extrapolate the available trend at present time to the recent past (back to 1960) and the near future (forward to 2030) and apply a steady decrease after 2030. The assumed future trend of organic chlorines reflects a plausible reduction in future demand for CHCl_3_ (raw material in HCFC-22 production) and CH_2_Cl_2_ (co-produced with CHCl_3_) once HCFC production is prohibited in developing countries from 2030. Projections of anthropogenic and biomass HCl for the 21st century are not available in the literature. Thus, the only available global anthropogenic emission inventory of HCl, proposed by ref. ^47^ for the year 1990, was scaled into the future based on the evolution of anthropogenic SO_2_ emission from RCP because anthropogenic HCl and SO_2_ share a common dominant source (coal burning). Biomass burning HCl reported in ref. ^47^ for the year 1990 is scaled with biomass burning CO for the common process (biomass burning) in RCP8.5 resulting in the future scaled emission that is lower than the original emission in 1990. In contrast, the scaled biomass burning HCl for RCP6.0 is higher than the original emission in 1990. To avoid introducing additional uncertainty, we decided to use constant biomass burning HCl emission strength for RCP6.0 (representative of the year 1990), which provides a conservative estimate of the Cl effect on CH_4_. The global average emission trends of all chlorine species used in the present study are shown in Supplementary Fig. 9. Future studies are recommended to investigate the evolution of reactive chlorine (particularly inorganic chlorine) emission in the past, at present, and in the future. Note that we conducted a sensitivity simulation with a higher emission of anthropogenic chlorine (continuously increasing CH_2_Cl_2_ from 2030 to 2100, constant C_2_Cl_4_ from 2030 to 2100, constant anthropogenic HCl from 1960 to 2100, scaled biomass burning HCl that increases from 1960 to 2100 following the RCP6.0 scenario) which leads to very similar changes in CH_4_ loss, lifetime, burden, and radiative forcing compared to the results reported in the main text. This confirms that while the uncertainties in emissions strengths of HCl might be relevant on a local to regional scale, its influence on the global changes of CH_4_ burden and radiative forcing is not significant.

### CESM model evaluation

The estimated atmospheric CH_4_ burdens in our simulations (Supplementary Table 2) are within the range of the reported estimates using various methods^4,5^. We have also evaluated the simulated CH_4_ mixing ratios with the available observations at the surface (Supplementary Fig. 10), and our results show that the simulated present time global CH_4_ levels (an average of 1732 ± 53 ppbv in noHAL_6.0, 1834 ± 50 ppbv in HAL_6.0, 1840 ± 92 ppbv in noHAL_8.5, and 1948 ± 93 ppbv in HAL_8.5) capture the range and magnitude of the observations by the NOAA network (1835 ± 69 ppbv)^48^.

Our simulated global tropospheric OH concentrations for the noHAL_6.0, HAL_6.0, noHAL_8.5, and HAL_8.5 cases are 1.04 × 10^6^, 9.78 × 10^5^, 9.29 × 10^5^, and 8.80 × 10^5^ # cm^−3^, respectively, averaged in the entire 21st century, and 1.07 × 10^6^, 1.01 × 10^6^, 1.05 × 10^6^, 9.81 × 10^5^ # cm^−3^, respectively, averaged in 2000–2019 period. These values are in good agreement with the reported observations and previous modeling studies of ~1.0 × 10^6^ # cm^−3^ for the present day (e.g., ref. ^23^ and the reference therein).

The evaluation of natural halogen species and routine atmospheric composition (e.g., O_3_) have been reported to reproduce the available observations in previous CESM studies^27,37,38,43,45,49^. Here, we further evaluate the model performance for anthropogenic chlorine species by comparing them with a compendium of observations reported in the recent past (Supplementary Fig. 11). The comparison of the simulated and observed surface HCl (compiled by ref. ^7^) indicates that our model captures the magnitude and spatial variation of HCl observations. The predicted CH_2_Cl_2_, the dominant anthropogenic organic chlorine very short-lived species, also reproduces the observed abundance and variation at the surface (https://gml.noaa.gov/aftp/data/hats/solvents/CH2Cl2/flasks/; personal communication to Stephen A. Montzka), with an average of 38.2 ± 20.7 pptv for observation and 37.9 ± 20.7 pptv for simulation, as well as in the troposphere (ref. ^50^).

## Supplementary information

Supplementary Information

## Data Availability

The CESM data generated in this study have been deposited in the Mendeley Data (https://data.mendeley.com/datasets/tjtpry4mgh/1).

## Code availability

The software code for the CESM model is available from http://www.cesm.ucar.edu/models/.
