# Supplementary material for: Reactive halogens increase the global methane lifetime and radiative forcing in the 21st century
Source: Nat Commun. 2022 May 19;13:2768. doi: 10.1038/s41467-022-30456-8 (PMC9120080; doi:10.1038/s41467-022-30456-8)
Supplement: Supplementary file 1 — Supplementary Information [file 41467_2022_30456_MOESM1_ESM.pdf]

Supplementary Information:

Reactive halogens increase the global methane lifetime and radiative forcing in the 21st century

Qinyi Li[1,*], Rafael P. Fernandez[2], Ryan Hossaini[3], Fernando Iglesias-Suarez[1,4], Carlos A. Cuevas[1], Eric C. Apel[5], Douglas E. Kinnison[5], Jean-François Lamarque[6], and Alfonso Saiz-Lopez[1,*]

[1] Department of Atmospheric Chemistry and Climate, Institute of Physical Chemistry Rocasolano, CSIC, Madrid 28006, Spain;

[2] Institute for Interdisciplinary Science (ICB), National Research Council (CONICET), FCEN-UNCuyo, Mendoza, Argentina;

[3] Lancaster Environment Centre, Lancaster University, Lancaster, UK;

[4] Deutsches Zentrum für Luft- und Raumfahrt (DLR), Institut für Physik der Atmosphäre, Oberpfaffenhofen, Germany;

[5] Atmospheric Chemistry Observations & Modeling Laboratory, National Center for Atmospheric Research, Boulder, CO, USA;

[6] Climate and Global Dynamics Laboratory, National Center for Atmospheric Research, Boulder, CO, USA.

[*] Corresponding author: Alfonso Saiz-Lopez (a.saiz@csic.es); Qinyi Li (qli@iqfr.csic.es).

Contents:

Supplementary Figures: Supplementary Fig. 1 to 11.

Supplementary Tables: Supplementary Table 1 to 3.

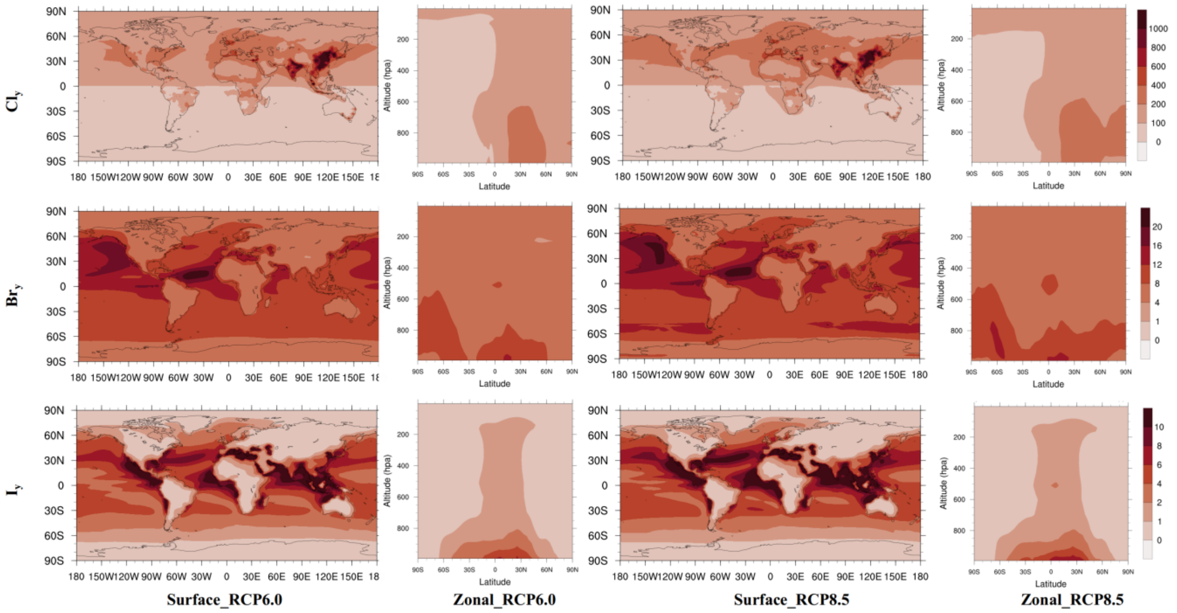

Supplementary Figure 1 | Simulated changes (difference between HAL and noHAL scenarios) in RHS (Cl_y, Br_y, and I_y) mixing ratios (pptv) for RCP6.0 (left) and RCP8.5 (right) scenarios in the 21st Century. Both surface and zonal average results are shown. The marked difference in Cl_y between the northern and southern hemispheres is because the source of Cl_y is dominated by anthropogenic emissions which are much higher in the northern hemisphere (particularly in the northern mid-latitude land) than in the southern hemisphere. The increase of bromine and iodine species is confined particularly in the tropical lower troposphere.

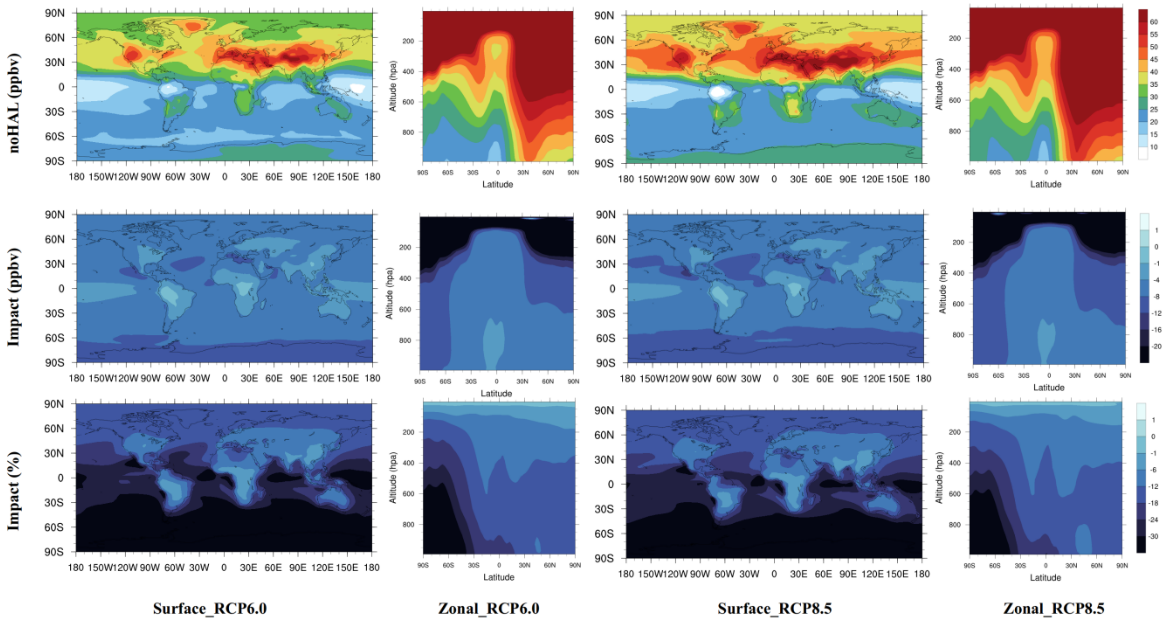

Supplementary Figure 2 | Predicted O_3 mixing ratios (ppbv) in noHAL case and their changes (ppbv and %) due to halogens for RCP6.0 (left) and RCP8.5 (right) scenarios in the 21st Century. Both surface and zonal average results are shown.

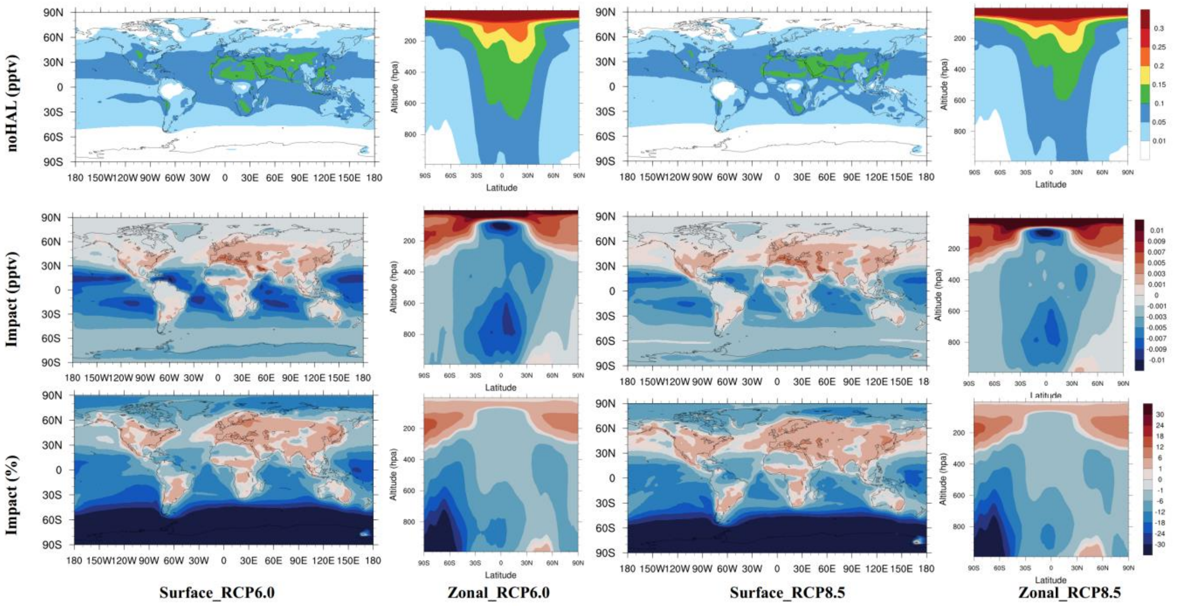

Supplementary Figure 3 | Predicted OH radical mixing ratios (pptv) in noHAL case and their changes (pptv and %) due to halogens for RCP6.0 (left) and RCP8.5 (right) scenarios in the 21st Century. Both surface and zonal average results are shown.

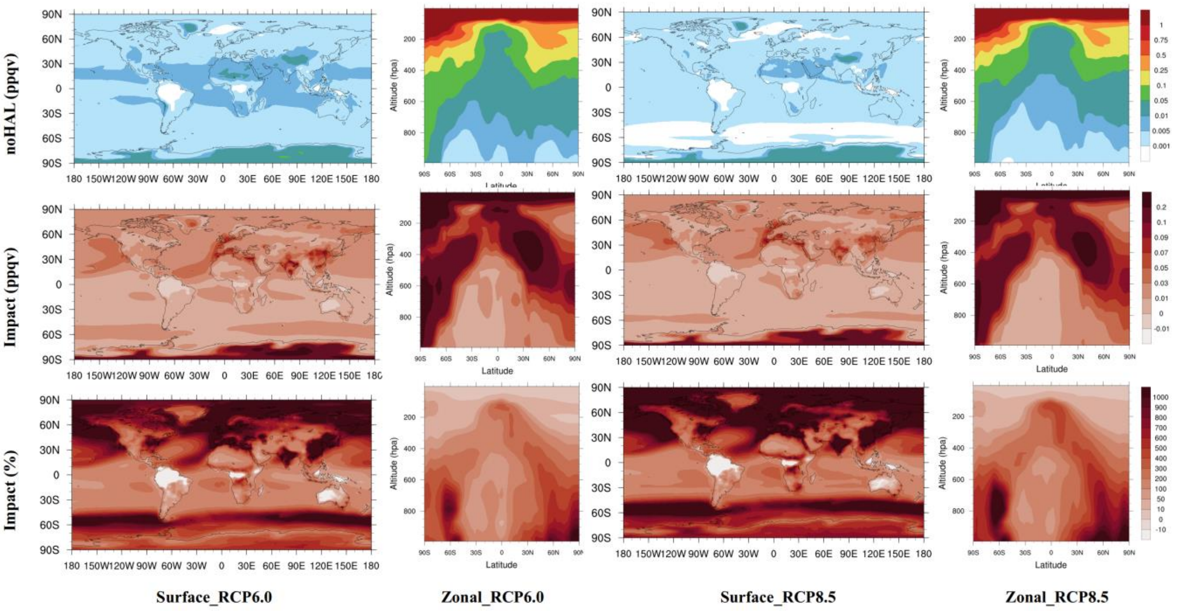

Supplementary Figure 4 | Predicted Cl atom mixing ratios (ppqv) in noHAL case and their changes (ppqv and %) due to halogens for RCP6.0 and RCP8.5 scenarios in the 21st Century. Both surface and zonal average results are shown.

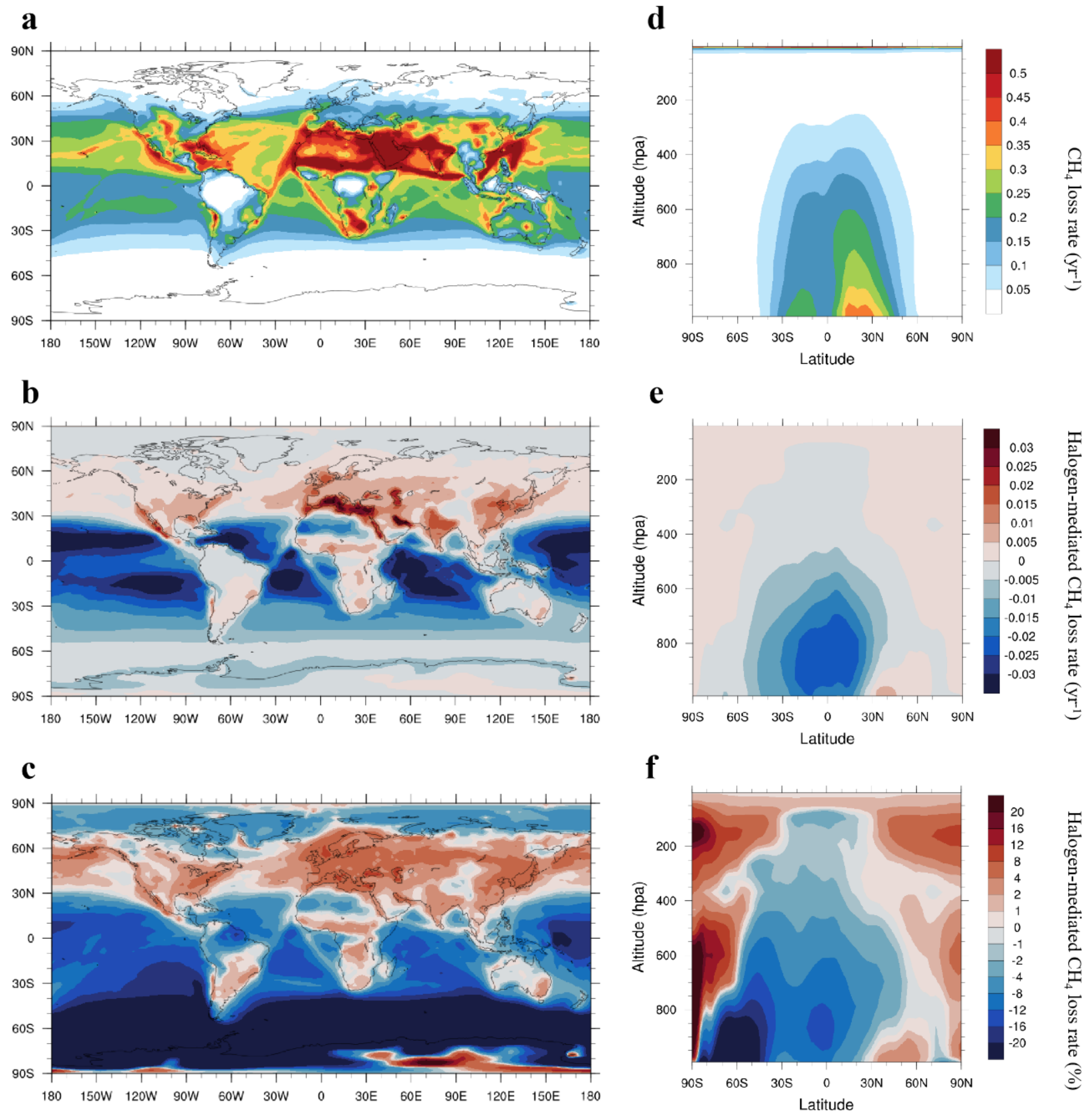

Supplementary Figure 5 | Spatial patterns of halogen-mediated change in CH_4 loss rate averaged during the 21st century for the RCP8.5 scenario. a surface CH_4 loss (yr^{-1}) without halogens (noHAL); **b** Halogen-mediated changes in CH_4 loss (yr^{-1}) at the surface; **c** same as **b** but in percentage; **d** Zonal distribution of CH_4 loss (yr^{-1}) without halogens (noHAL) showing the largest CH_4 loss near-surface level in tropics; **e** Zonal distribution of halogen-mediated change in CH_4 loss (yr^{-1}); **f** same as **e** but in percentage. Results for the RCP6.0 scenario are shown in Figure 2.

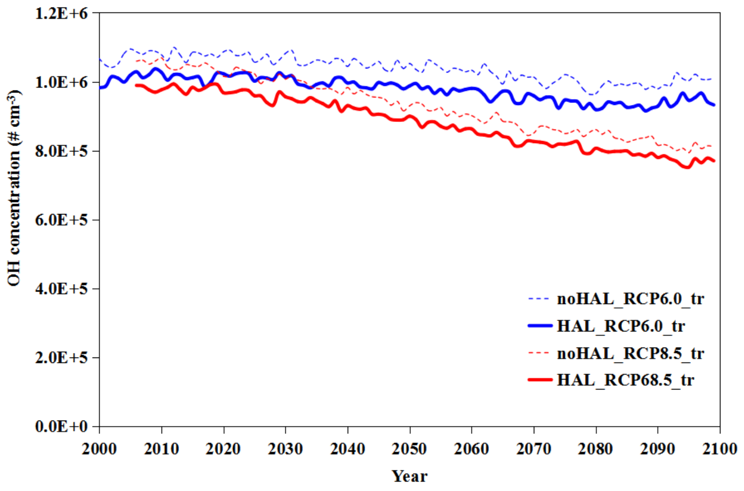

Supplementary Figure 6 | The variation of global tropospheric OH concentration (# cm^{-3}) in noHAL and HAL cases in both RCP6.0 and RCP8.5 scenarios in the 21st century.

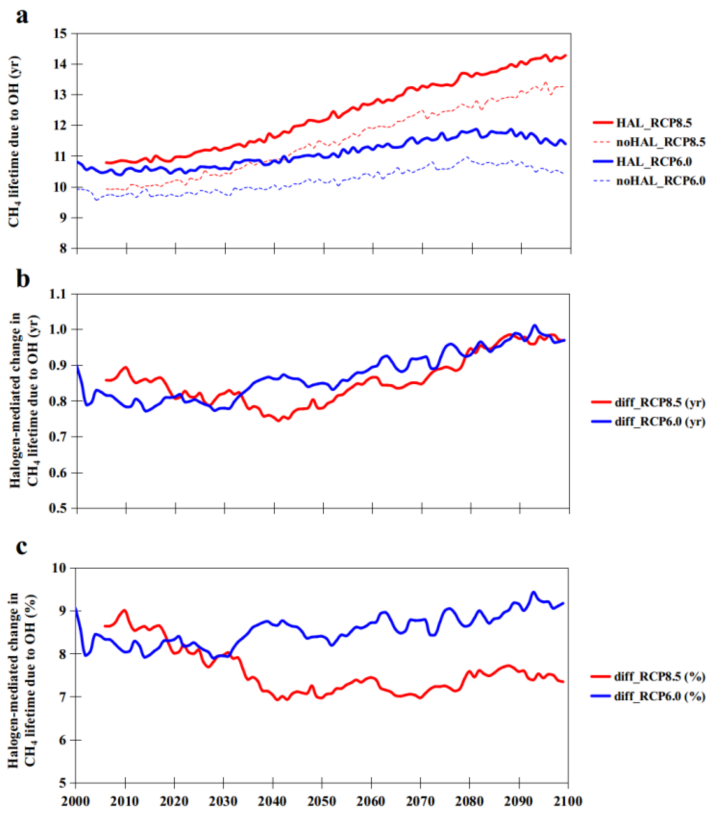

Supplementary Figure 7 | Halogen-mediated changes in CH_4 lifetime with respect to OH in the 21st century for RCP6.0 and RCP8.5 scenarios. **a** Global CH_4 chemical lifetime (yr) with (HAL, solid) and without (noHAL, dashed) halogens; **b** halogen-mediated change in CH_4 lifetime in absolute term (yr); **c** the same as **b** but in percentage. Note that the halogen-mediated changes are calculated with a moving average of 10 years (approximately the lifetime of CH_4 in the atmosphere). The total CH_4 chemical lifetime is shown in Figure 4. Note that the results for RCP8.5 are only shown from 2006 to 2100 and the results before 2006 are identical to those in RCP6.0.

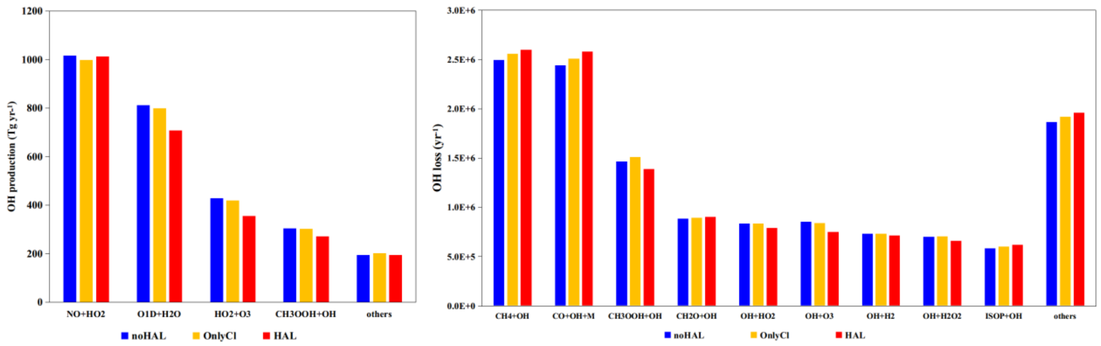

Supplementary Figure 8 | Budget analysis of global OH production (left, Tg yr^{-1}) and loss (right, yr^{-1}) in noHAL, OnlyCl, and HAL cases averaged in the entire 21st century. Note that the OH loss is normalized with its burden.

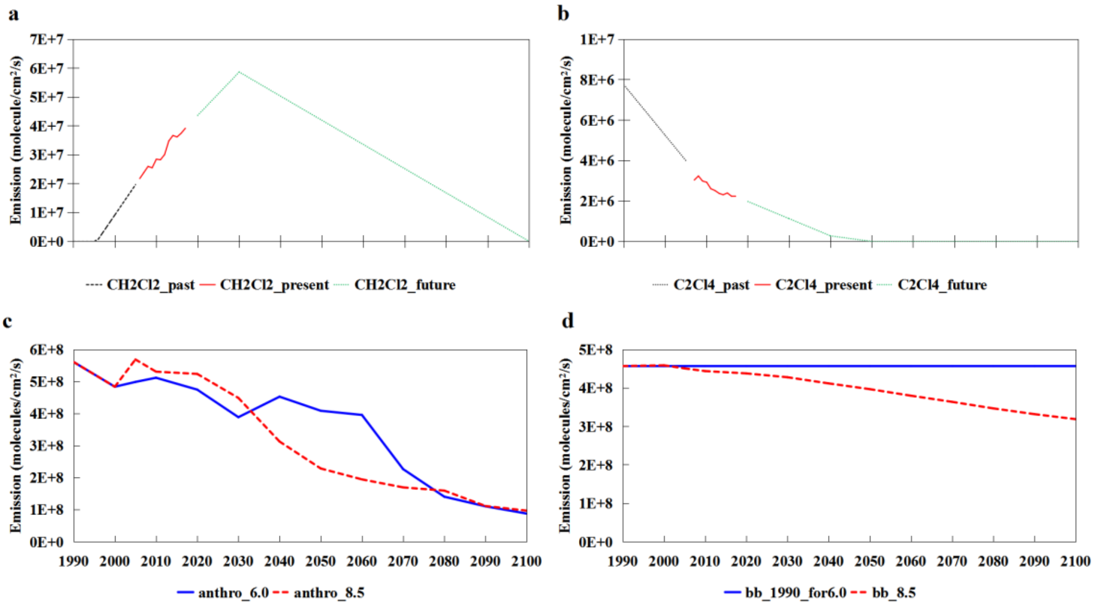

Supplementary Figure 9 | The emission trend of (**a**) anthropogenic CH_2Cl_2, (**b**) anthropogenic C_2Cl_4, (**c**) anthropogenic HCl, and (**d**) biomass burning HCl used in the present study.

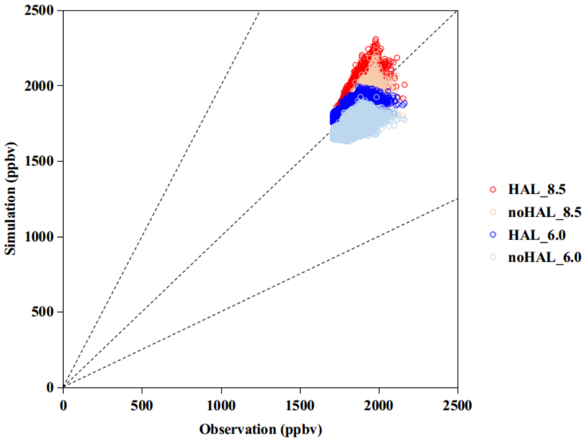

Supplementary Figure 10 | Comparison of simulated surface CH_4 volume mixing ratio with global surface observations from NOAA. The dotted lines represent 1:2, 1:1, and 2:1 ratios.

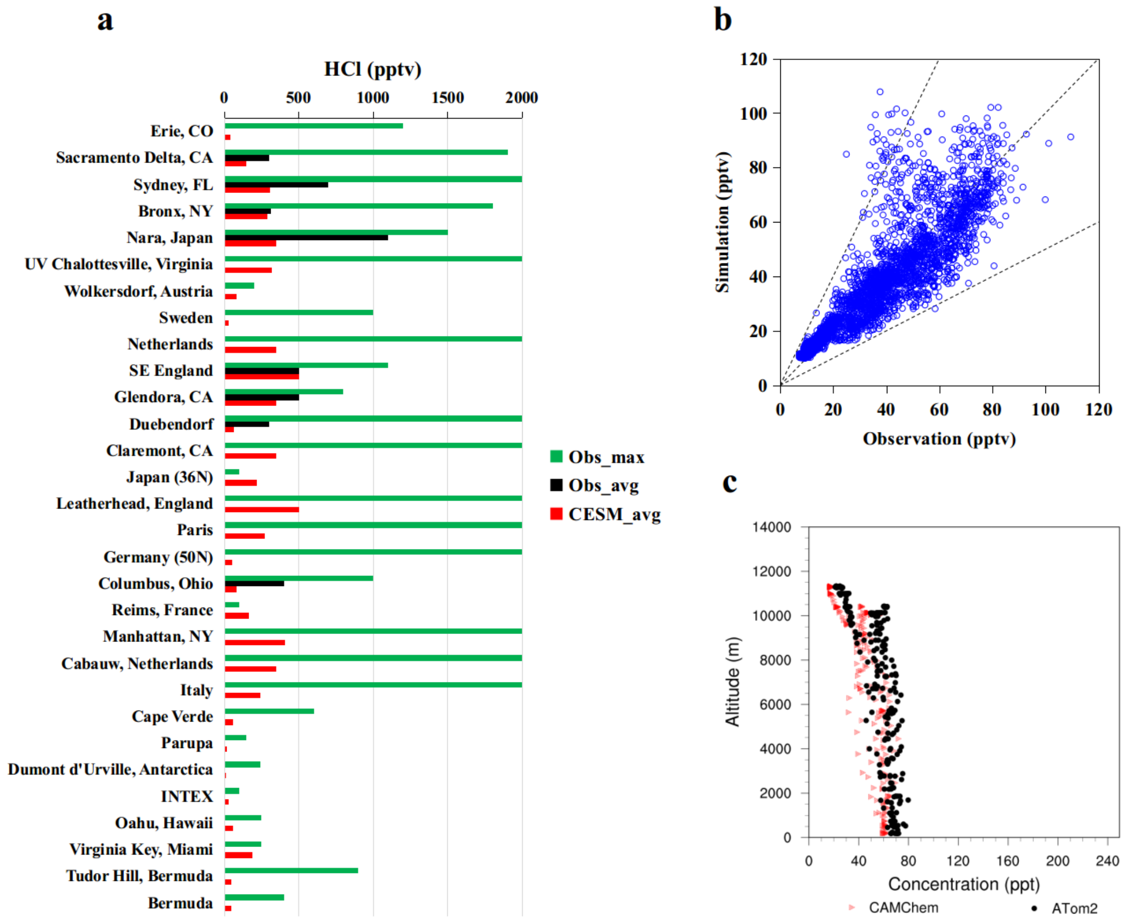

Supplementary Figure 11 | (a) Comparison of simulated HCl with surface observation, (**b**) comparison of simulated CH_2Cl_2 and surface observation (the dotted lines are 1:2, 1:1, and 2:1 ratios), and (**c**) comparison of simulated CH_2Cl_2 and flight observation during ATom campaign. Note that the observed maximum of HCl at the following sites are larger than 2000 pptv: Sydney, FL (5600 pptv), UV Chalottesville, Virginia (2800 pptv), Netherlands (3000 pptv), Leatherhead, England (3800 pptv), Paris (5000 pptv), Manhattan, NY (9000 pptv), Italy (2500 pptv).

Supplementary Table 1 | Design of simulation scenarios

Scenario	Routine emissions	Natural VSL	Anthropogenic HCl	Biomass burning HCl	CH_2Cl_2	Other organic chlorine
**noHAL_6.0**	RCP6.0	-	-	-	-	-
**HAL_6.0**	RCP6.0	Iglesias-Suarez et al. (2020)	Scaling with anthropogenic SO_2 in RCP6.0	Fixed from 1960 to 2100 using Keene et al., 1999 (RCEI)	Variation from 1960 to 2030; Decrease afterwards	Variation from 1960 to 2030; Decrease to natural value by 2100.
**OnlyCl_6.0**	RCP6.0	Chlorine species in Iglesias-Suarez et al. (2020)	Scaling with anthropogenic SO_2 in RCP6.0	Fixed from 1960 to 2100 using Keene et al., 1999 (RCEI)	Variation from 1960 to 2030; Decrease afterwards	Variation from 1960 to 2030; Decrease to natural value by 2100.
**noHAL_8.5**	RCP8.5	-	-	-	-	-
**HAL_8.5**	RCP8.5	Iglesias-Suarez et al. (2020)	Scaling with anthropogenic SO_2 in RCP8.5	Scaling with biomass burning CO in RCP8.5	Variation from 1960 to 2030; Decrease afterwards	Variation from 1960 to 2030; Decrease to natural value by 2100.

Note: the description of the model design is in Methods.

Supplementary Table 2 | Statistics of global CH_4 burden, source, and sink at present, in the future, and in the 21st century.

	Burden (Tg)				Source (Tg/yr)						Sink (Tg/yr)											
	RCP6.0		RCP8.5		RCP6.0			RCP8.5			RCP6.0						RCP8.5					
											noHAL			HAL			noHAL			HAL		
	noHAL [a]	HAL [b]	noHAL [c]	HAL [d]	ANT [e]	BB [f]	NAT [g]	ANT [h]	BB [i]	NAT [j]	OH [k]	Cl [l]	Str [m]	OH [n]	Cl [o]	Str [p]	OH [q]	Cl [r]	Str [s]	OH [t]	Cl [u]	Str [v]
**Present (2000-2019) [w]**	4714.7	5000.6	4994.9	5299.2	280.8	25.7	191.0	331.8	24.7	191.0	483.5	2.7	27.1	473.9	9.6	29.0	499.4	2.7	28.1	488.8	9.9	32.3
**Future (2080-2099) [x]**	5116.0	5509.0	11771.2	12423.6	250.3	30.1	191.0	837.0	16.4	191.0	479.7	1.9	26.3	473.1	5.7	28.5	906.3	1.8	62.6	889.7	8.0	66.7
**21st Century [y]**	4971.9	5302.8	8381.9	8825.9	287.4	27.4	191.0	582.3	21.0	191.0	487.3	2.3	26.8	478.6	7.9	28.8	714.3	2.2	45.0	700.0	9.3	47.8

[a] Global CH_4 burden (Tg/yr) in the RCP6.0 scenario in the noHAL case;
[b] Global CH_4 burden (Tg/yr) in the RCP6.0 scenario in the HAL case;
[c] Global CH_4 burden (Tg/yr) in the RCP8.5 scenario in the noHAL case;
[d] Global CH_4 burden (Tg/yr) in the RCP8.5 scenario in the HAL case;
[e] Global CH_4 source (Tg/yr) in the RCP6.0 scenario from anthropogenic emission;
[f] Global CH_4 source (Tg/yr) in the RCP6.0 scenario from biomass burning emission;
[g] Global CH_4 source (Tg/yr) in the RCP6.0 scenario from natural emission;
[h] Global CH_4 source (Tg/yr) in the RCP8.5 scenario from anthropogenic emission;
[i] Global CH_4 source (Tg/yr) in the RCP8.5 scenario from biomass burning emission;
[j] Global CH_4 source (Tg/yr) in the RCP8.5 scenario from natural emission;
[k] Global CH_4 sink (Tg/yr) in the RCP6.0 scenario in the noHAL case via tropospheric OH;
[l] Global CH_4 sink (Tg/yr) in the RCP6.0 scenario in the noHAL case via tropospheric Cl;
[m] Global CH_4 sink (Tg/yr) in the RCP6.0 scenario in the noHAL case via stratospheric processes, including stratospheric OH, Cl, and O^1D;
[n] Global CH_4 sink (Tg/yr) in the RCP6.0 scenario in the HAL case via tropospheric OH;
[o] Global CH_4 sink (Tg/yr) in the RCP6.0 scenario in the HAL case via tropospheric Cl;
[p] Global CH_4 sink(Tg/yr) in the RCP6.0 scenario in the HAL case via stratospheric processes, including stratospheric OH, Cl, and O^1D;
[q] Global CH_4 sink (Tg/yr) in the RCP8.5 scenario in the noHAL case via tropospheric OH;
[r] Global CH_4 sink (Tg/yr) in the RCP8.5 scenario in the noHAL case via tropospheric Cl;
[s] Global CH_4 sink (Tg/yr) in the RCP8.5 scenario in the noHAL case via stratospheric processes, including stratospheric OH, Cl, and O^1D;
[t] Global CH_4 sink (Tg/yr) in the RCP8.5 scenario in the HAL case via tropospheric OH;
[u] Global CH_4 sink (Tg/yr) in the RCP8.5 scenario in the HAL case via tropospheric Cl;
[v] Global CH_4 sink (Tg/yr) in the RCP8.5 scenario in the HAL case via stratospheric processes, including stratospheric OH, Cl, and O^1D;
[w] Values averaged for the present time (2000 to 2019);
[x] Values averaged for the future (2080 to 2099);
[y] Values averaged for the 21st century (2000 to 2099);

Supplementary Table 3. Global methane burden (Tg) in the year 2000 in the sensitivity cases with (HAL) and without (noHAL) halogens applying different spin-up periods (10, 20, 30, and 40 years).

	CH_4 burden	difference		CH_4 burden	difference
noHAL_10	4114	-	HAL_10	4244	-
noHAL_20	4576	11.2% [a]	HAL_20	4797	13.0% [d]
noHAL_30	4769	4.2% [b]	HAL_30	5028	4.8% [e]
noHAL_40	4840	1.5% [c]	HAL_40	5116	1.8% [f]

[a] The difference is calculated as (nohal_20 - nohal_10)×100/nohal_10.
[b] The difference is calculated as (nohal_30 - nohal_20)×100/nohal_20.
[c] The difference is calculated as (nohal_40 - nohal_30)×100/nohal_30.
[d] The difference is calculated as (hal_20 - hal_10)×100/hal_10.
[e] The difference is calculated as (hal_30 - hal_20)×100/hal_20.
[f] The difference is calculated as (hal_40 - hal_30)×100/hal_30.
